# Development of I_KATP_ Ion Channel Blockers Targeting Sulfonylurea Resistant Mutant K_IR_6.2 Based Channels for Treating DEND Syndrome

**DOI:** 10.3389/fphar.2021.814066

**Published:** 2022-01-14

**Authors:** Marien J. C. Houtman, Theres Friesacher, Xingyu Chen, Eva-Maria Zangerl-Plessl, Marcel A. G. van der Heyden, Anna Stary-Weinzinger

**Affiliations:** ^1^ Department of Medical Physiology, Division of Heart and Lungs, University Medical Center Utrecht, Utrecht, Netherlands; ^2^ Department of Pharmaceutical Sciences, Division of Pharmacology and Toxicology, University of Vienna, Vienna, Austria

**Keywords:** SUR2, DEND syndrome, betaxolol, travoprost, patch clamp, KIR6.2, molecular dynamics

## Abstract

**Introduction:** DEND syndrome is a rare channelopathy characterized by a combination of developmental delay, epilepsy and severe neonatal diabetes. Gain of function mutations in the *KCNJ11* gene, encoding the K_IR_6.2 subunit of the I_KATP_ potassium channel, stand at the basis of most forms of DEND syndrome. In a previous search for existing drugs with the potential of targeting Cantú Syndrome, also resulting from increased I_KATP_, we found a set of candidate drugs that may also possess the potential to target DEND syndrome. In the current work, we combined Molecular Modelling including Molecular Dynamics simulations, with single cell patch clamp electrophysiology, in order to test the effect of selected drug candidates on the K_IR_6.2 WT and DEND mutant channels.

**Methods:** Molecular dynamics simulations were performed to investigate potential drug binding sites. To conduct *in vitro* studies, K_IR_6.2 Q52R and L164P mutants were constructed. Inside/out patch clamp electrophysiology on transiently transfected HEK293T cells was performed for establishing drug-channel inhibition relationships.

**Results:** Molecular Dynamics simulations provided insight in potential channel interaction and shed light on possible mechanisms of action of the tested drug candidates. Effective I_KIR6.2/SUR2a_ inhibition was obtained with the pore-blocker betaxolol (IC_50_ values 27–37 μM). Levobetaxolol effectively inhibited WT and L164P (IC_50_ values 22 μM) and Q52R (IC_50_ 55 μM) channels. Of the SUR binding prostaglandin series, travoprost was found to be the best blocker of WT and L164P channels (IC_50_ 2–3 μM), while Q52R inhibition was 15–20% at 10 μM.

**Conclusion:** Our combination of MD and inside-out electrophysiology provides the rationale for drug mediated I_KATP_ inhibition, and will be the basis for 1) screening of additional existing drugs for repurposing to address DEND syndrome, and 2) rationalized medicinal chemistry to improve I_KATP_ inhibitor efficacy and specificity.

## Introduction

Potassium carrying K_ATP_ channels transduce the cellular metabolic status to electrophysiological properties in many cell types. In pancreatic beta-cells, high intracellular ATP levels, and low MgADP levels, inhibit K_ATP_ channel activity which slightly depolarizes the cells resulting in insulin release (Ashcroft, 2005). K_ATP_ channels are octameric protein complexes consisting of a tetrameric pore-forming K_IR_6.1 or K_IR_6.2 protein assembly, encoded by *KCNJ8* and *KCNJ11*, respectively, surrounded by a tetramer of SUR1 or SUR2A/B proteins, encoded by *ABCC8* and *ABCC9*, respectively (Hibino et al., 2010). Gain of function mutations in the underlying genes associate with diverse disease phenotypes affecting one or more tissues and organs, like transient or permanent neonatal diabetes (ND) and Cantú disease (Gloyn et al., 2004; Harakalova et al., 2012). Binding of ATP to the K_IR_6.2 subunit has an inhibitory effect, whilst binding of nucleotides to the SUR subunit activates the channel (Sikimic et al., 2019). Gain of function mutations decrease the ability of ATP to inhibit the channel, either directly by affecting the ATP binding site, or indirectly by increasing channel open probability, thereby decreasing ATP affinity (Ashcroft, 2005). There is a clear relation between the extent of diabetes causing *KCNJ11* gain of function strength and clinical phenotype. Whereas K_IR_6.2 channels harboring mild gain of function mutations result in transient or permanent ND, strong gain of function mutant K_IR_6.2 channels on the other hand associate with a complex phenotype of developmental delay, muscle weakness, dysmorphic features, epilepsy and neonatal diabetes, known as the DEND syndrome (Hattersley and Ashcroft, 2005). These strong gain of function mutations are generally of the indirect class.

Sulfonylurea type drugs, like tolbutamine and gliclazide, stimulate insulin secretion from beta-cells (Proks et al., 2002). Successful sulfonylurea therapy results in K_ATP_ channel inhibition, by direct and indirect mechanisms in which the pharmakon interacts with the SUR subunits, and thereby enhances ATP sensitivity of the K_IR_6.2 subunits and thus stimulate channel closure at lower intracellular ATP concentrations (De Wet and Proks, 2015). From a therapeutic perspective, the stronger gain of function mutations pose a pharmacological challenge since they are relatively resistant to sulfonylureas, and even in cases when the ND part of the phenotype is relieved, neurological symptoms are mostly less-, or non-sensitive to such treatment (Koster et al., 2005; Pearson et al., 2006). This is even worsened by difficulties of many existing and newly developed compounds to efficiently cross the blood-brain-barrier and reach therapeutic concentrations in the neural tissues (Ashcroft et al., 2017). Apart from this, some mutants associated with ND only, are also relatively resistant to sulfonylureas (Tammaro et al., 2008).

**FIGURE 1:**
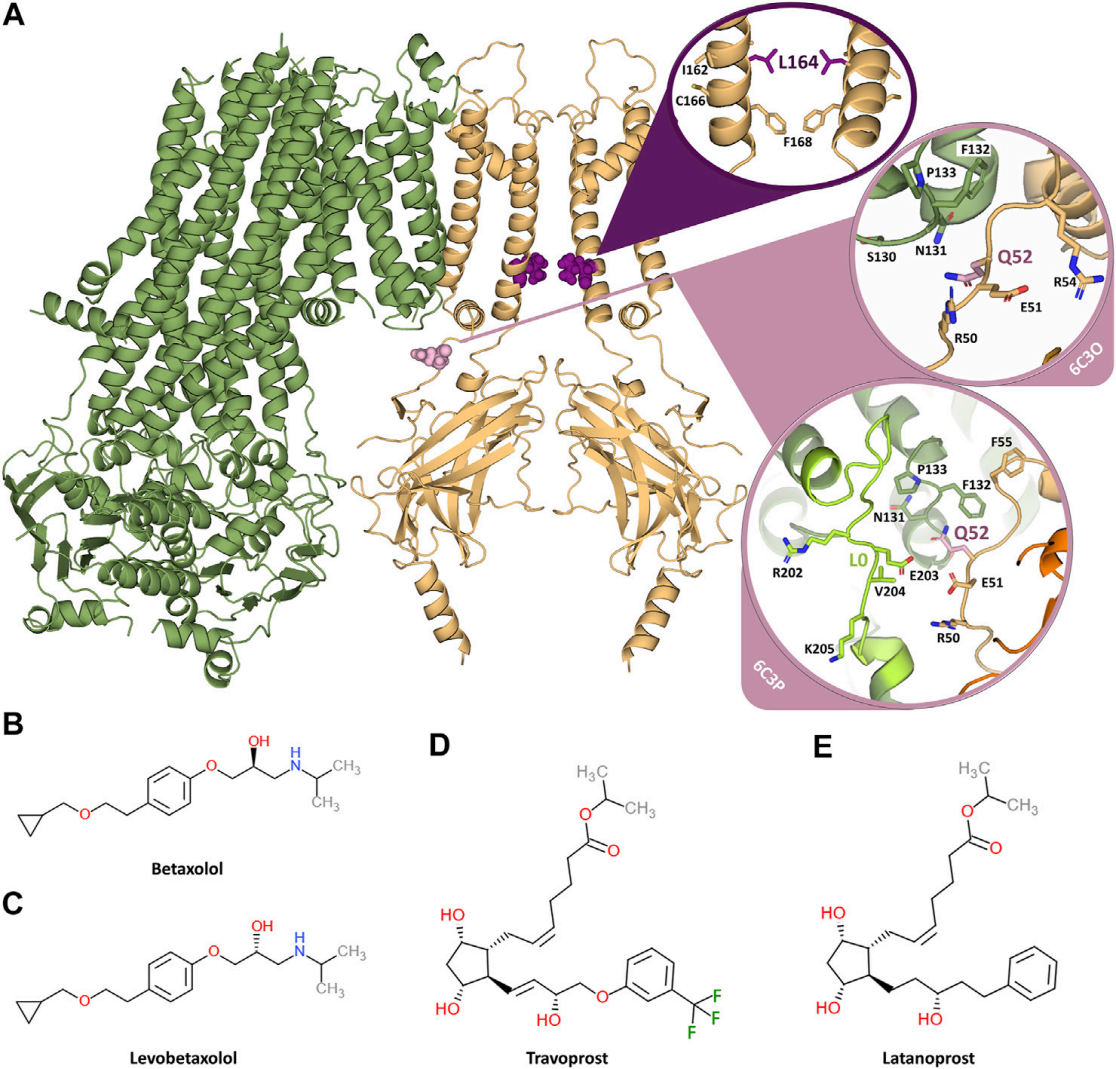
Overview of the K_ATP_ channel and the investigated inhibiting compounds. **(A)** Two opposing subunits of the K_IR_6.2 channel pore are shown in orange, one SUR1 subunit is shown in green. The two mutation sites L164 and Q52 are shown in purple and pink, respectively. The circles provide close-ups of the mutation sites. Since Q52 is located on the surface of K_IR_6.2 close to SUR1 and the orientation of the SUR1 subunits is different in the 6C3O and 6C3P structure, the close-ups of Q52 are shown for both structures. The loop L0, which comprises residues important for K_IR_-SUR coupling, is shown in light green for the 6C3P structure, while it is not modelled in the 6C3O structure. **(B)** Chemical structure of betaxolol. **(C)** Chemical structure of levobetaxolol. **(D)** Chemical structure of travoprost. **(E)** Chemical structure of latanoprost.

The *KCNJ11* mutation Q52R associates with DEND syndrome, although clinical phenotype and therapeutic options vary between patients (Gloyn et al., 2004; Shaw and Majzoub, 2009; Ioacara et al., 2017; Helmi and Hussain, 2020). The L164P mutation however associates with the “milder” phenotype of permanent ND (Flanagan et al., 2006). Both mutations alter channel kinetics in a similar fashion, that is an increased open probability in the absence of ligand, which at least for the Q52R mutation was independent of the SUR isoform (Tammaro et al., 2006, 2008). The position of the mutations is shown in Figure 1A. Importantly, both mutations render the resulting K_ATP_ channel rather insensitive to sulfonylureas like tolbutamine (Pearson, et al., 2006; Tammaro et al., 2008; Babiker et al., 2016; Hashimoto et al., 2017). Therefore, there is a need for new compounds with desired pharmacokinetic/pharmacodynamic properties that effectively inhibit DEND syndrome associated mutant K_ATP_ channel and additional sulfonylurea resistant K_ATP_ channel variants.

Previously, in a successful attempt to find potential inhibitors of Cantú Syndrome associated gain of function mutants, we set out to screen for potential K_IR_6.1 inhibiting compounds by applying Molecular Dynamics and pharmacophore modelling (Chen et al., 2019). This work resulted in a hit-list from DrugBank entries. Here, we expanded this in silico and *in vitro* work to K_IR_6.2 inhibiting compounds betaxolol, levobetaxolol, latanoprost and travoprost (for chemical structure, see Figures 1B–E) as well as glibenclamide, ethosuximide, fluvastatin, pazopanib and dinoprostone (for chemical structure see Supplementary Figure S1) focusing on Q52R and L164P gain of function mutations.

## Materials and Methods

### Constructs and Mutagenesis

Nucleotide mutations resulting in Q52R and L164P were made in pCMV6-K_IR_6.2 (mouse) expression construct (a generous gift from J.A. Sánchez-Chapula (Centro Universitario de Investigaciones Biomédicas de la Universidad de Colima) using the Quickchange Site-Directed Mutagenesis kit (Agilent Technologies), by the use of custom made primers (Merck): (Q52R forward primer: caa​gaa​cat​ccg​aga​gcg​ggg​ccg​ctt​cct​gca​ag, reverse primer: ctt​gca​gga​agc​ggc​ccc​gct​ctc​gga​tgt​tct​tg; L164P forward primer: gat​caa​cgc​cat​cat​gcc​ggg​ctg​cat​ctt​cat​g, reverse primer: cat​gaa​gat​gca​gcc​cgg​cat​gat​ggc​gtt​gat​c). Mutations were verified using Sanger based sequencing (Macrogen Europe B.V.) and SnapGene analysis software (GSL Biotech LLC).

### Compounds

Stock solutions were prepared as follows: Barium (Merck, cat. nr. 1.01719) at 1 M in H_2_O, sterile filtered; betaxolol (Merck, cat. nr. 1069903) at 0.1 M in H_2_O; dinoprostone (Merck, cat. nr. D2250000) at 0.1 M in EtOH; ethosuximide (Merck, cat. nr. E7138) at 0.1 M in EtOH; fluvastatin (Merck, cat. nr. SML0038) at 10 mM in H_2_O; glibenclamide (Merck, cat. nr. G0639) at 0.1 M in DMSO; latanoprost (Merck, cat. nr. PHR 1884) at 1 mM in EtOH; levobetaxolol (Cayman Chemical, cat. nr. 33435-250) at 0.1 M in H_2_O; travoprost (Toronto Research Chemicals, cat. nr. T715600) at 10 mM in DMSO. Barium was stored at 4°C and all other compounds were stored at −20°C until use.

### Inside-Out I_KATP_ Electrophysiology

HEK293T cells were cultured in DMEM medium (Lonza, cat. nr. 12–733F) and seeded on glass coverslips, coated with gelatin (Merck, cat. nr. G1890). After cell adherence, polyethylenimine (Polysciences, cat. nr. 23966) transfection was performed using either WT or mutant pCMV6-K_IR_6.2 in combination with pCMV6-SUR2A and pEGFP1 (0.16, 0.16 and 0.08 μg, respectively). I_KATP_ measurements were performed essentially as described before (Houtman et al., 2019). Briefly, glass capillary pipettes (Harvard Apparatus, cat. nr. 30–0,040) filled with pipette solution (145 mM KCl, 1 mM CaCl_2_, 1 mM MgCl_2_, 5 mM HEPES, pH 7.40 KOH) were introduced in a temperature controlled perfusion-chamber (CellMicroControls) filled with bath solution (131 mM KCl, 1 mM EGTA, 7.2 mM K_2_HPO_4_, 2.8 mM KH_2_PO_4_, 1 mM MgCl_2_, pH 7.20 KOH). After giga-seal formation, the pipette was pulled up and after brief air exposure inside-out patches were formed. Using an AxoPatch 200B amplifier and Clampex 10 software (Molecular Devices) a ramp protocol was run with holding potentials ranging from −100 mV to +100 mV. Inward and outward I_KATP_ current levels at −80 mV and +50 mV were determined using Clampfit 10 software (Molecular Devices). All measurements were performed at room temperature (22°C). Data and statistical analysis (one-way ANOVA and t test) were performed using GraphPad Prism 8 software (GraphPad Software LLC). Data are presented as mean ± s.e.m.

### Molecular Dynamics (MD) Simulations

Molecular modelling of drug interactions with the K_ATP_ channel were carried out using coarse-grained and classical MD simulations. An overview of the simulation runs is given in Supplementary Table S1.

#### Coarse-Grained Molecular Dynamics Simulations

The quatrefoil (PDB: 6C3O) and propeller (PDB:6C3P) structures (Lee et al., 2017) were set up with the Martini bilayer maker of the CHARMM-GUI web service (Jo et al., 2008; Qi et al., 2015; Hsu et al., 2017). For the coarse-grained MD simulations without SUR, the K_IR_6.2 pore was extracted from the K_ATP_ channel (PDB: 6C3O) and used as starting structure. Simulations were carried out based on the Martini2.2 coarse-grained model (Marrink et al., 2007; Monticelli et al., 2008), implementing the global elastic network (Periole et al., 2009). The channels were embedded in a palmitoyloleoylphosphatidylcholine (POPC) membrane and solvated with the non-polarizable water model in a 150 mM KCl solution. Simulations were carried out with Gromacs 2018 and 2020 (Abraham et al., 2015). Temperature was coupled to 310 K using the v-rescale thermostat (Bussi et al., 2007) with a coupling constant of 0.1 ps. The pressure was kept constant semi-isotropically at 1 bar with the Parrinello-Rahman barostat (τ = 2 ps) (Parrinello and Rahman, 1981). Simulations with betaxolol were computed with an integration step of 2 fs, whereas simulations with the prostaglandins required an integration step of 1 fs in order to increase the stability of the system. Latanoprost, travoprost and betaxolol were parametrized as described on the Martini homepage (Martini, 2021). The topologies of the ligands can be found in Supplementary Table S2.

#### All-Atom Molecular Dynamics Simulations

All-atom MD simulations with K_IR_6.2 and betaxolol were performed as described previously (Chen et al., 2019, PDB:6C3O). For this purpose, betaxolol was docked into the binding sites, which were identified in the coarse-grained MD simulations, using the GOLD software (Verdonk et al., 2003) with ChemPLP scoring function (Korb et al., 2009). We used the Gromacs 2018 (Abraham et al., 2015) software with the Amber99sb force field (Hornak et al., 2006) and embedded the K_IR_6.2 channel pores in a palmitoyloleoylphosphatidylcholine (POPC) lipid bilayer [Berger lipids parameters (Berger et al., 1997)] with four PIP_2_ molecules bound to the channel. Ligand parameters for PIP_2_ and betaxolol were obtained using the Hartree-Fock geometry optimization with the 6-31G* basis set (Frisch et al., 2013) and the antechamber tools (Wang et al., 2004; Wang et al., 2006). The SPCE water model (Berendsen et al., 1987; Kusalik and Svishchev, 1994) was used and 150 mM KCl were added to the solvent. Five K^+^ ions were placed in the selectivity filter at sites S0 to S4. 2 times 1 μs MD simulations and 9 times 350 ns were run, including the four main identified betaxolol binding sites, obtained from coarse-grained simulations. The topology of betaxolol can be found in Supplementary Table S2.

#### Analysis and Visualization

The simulations were analyzed using the analysis tools of Gromacs (Abraham et al., 2015). Furthermore, the binding sites observed in the coarse-grained MD simulations were analyzed with the Python package MDAnalysis (Michaud-Agrawal et al., 2011; Gowers et al., 2016). In-house Python 3 scripts were used for plotting. The molecular structures were visualized with PyMol (PyMol, 2016) and VMD (Humphrey et al., 1996).

## Results

### Pharmacological Validation Q52R and L164P Mutations

**FIGURE 2:**
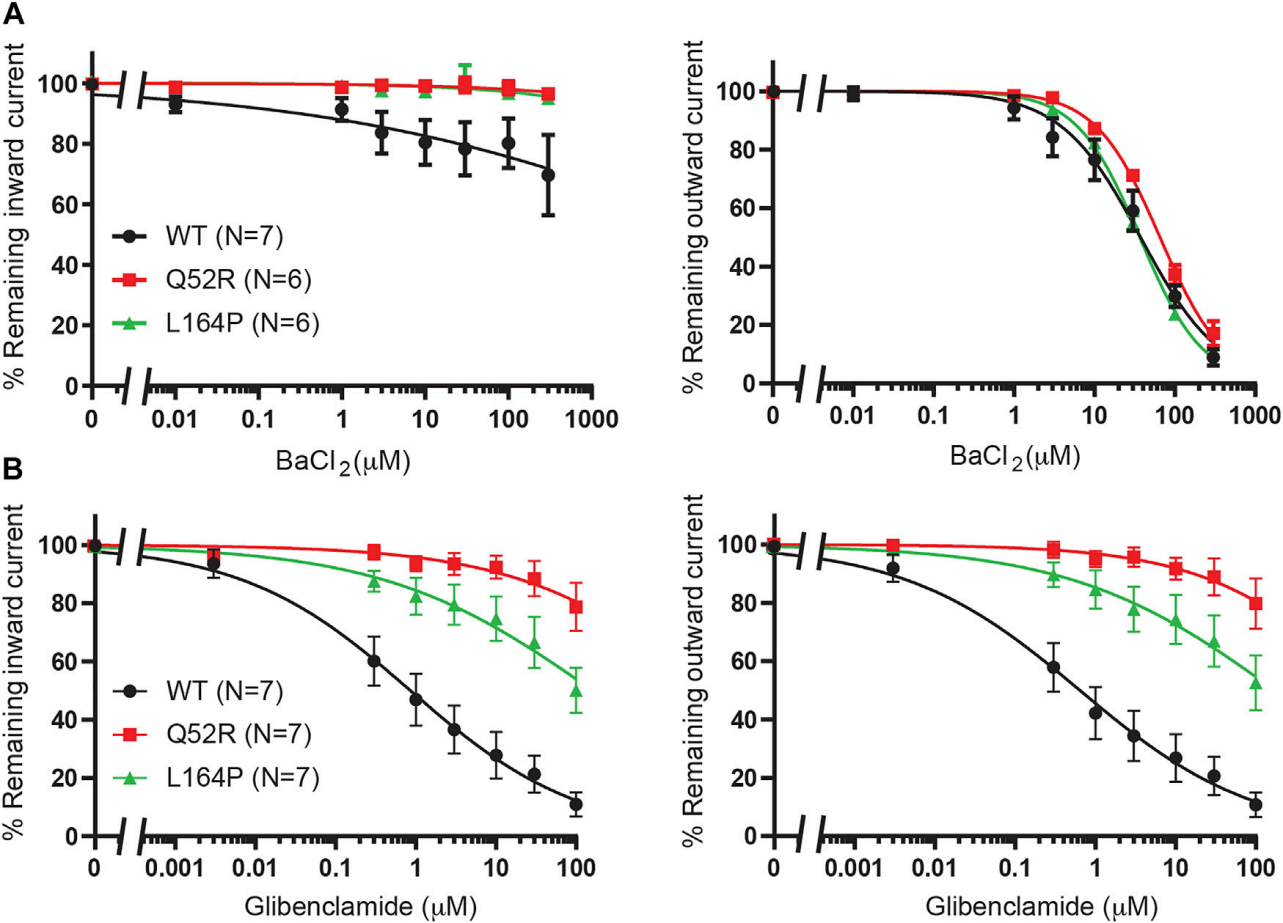
Pharmacological validation of Q52R and L164P K_IR_6.2 mutations. IC_50_ curves of K_IR_6.2/SUR2a inward (at −80 mV), left panels, and outward (at +50 mV), right panels, current inhibition in response to increasing concentrations of **(A)** BaCl_2_ and **(B)** glibenclamide. Wildtype K_IR_6.2/SUR2a serves as control. Data were fitted with Hill equation to estimate the IC_50_ values. Data are shown as mean ± SEM. N depicts number of measurements on independent inside-out patches.

We first created the K_IR_6.2 Q52R and L164P mutant channels and subsequently tested these using inside-out recordings on membrane patches from transiently transfected HEK293T cells in the absence of MgATP/ADP. Barium, as a positive control, dose-dependently inhibited outward current (at +50 mV) equally well for mutant channels as for WT channels (IC_50_ 37.4 ± 4.9 μM, Hill coefficient −0.87 (WT) vs. 65.2 ± 3.5 μM, Hill coefficient −1.1 (Q52R) (n.s.) and 36.5 ± 1.2 μM, Hill coefficient −1.1 (L164P) (n.s.), whereas inward current (at −80 mV) was less potently inhibited compared to the outward current for WT and not inhibited for Q52R and L164P channels (Figure 2A). The sulfonylurea drug glibenclamide potently inhibited inward and outward currents of the WT channels (IC_50_ 0.9 ± 0.3 μM, Hill coefficient −0.41 and 0.6 ± 0.2 μM, Hill coefficient −0.40 for inward and outward currents respectively), whereas Q52R channels were insensitive for glibenclamide mediated block (<10% block at 10 μM) (Figure 2B). The L164P channels were less potently inhibited compared to WT channels (approx. 20–25% block at 10 μM). These data demonstrate the expected behavior of the mutant channels in relation to drug sensitivity of the WT channels.

We next expanded our previous findings, in which we demonstrated that betaxolol (n^o^ 5 on Chen et al., 2019 hit list) and Travoprost (n^o^ 7) were able to inhibit I_KIR6.2/SUR2a_ outward currents.

### Betaxolol and Levobetaxolol

**FIGURE 3:**
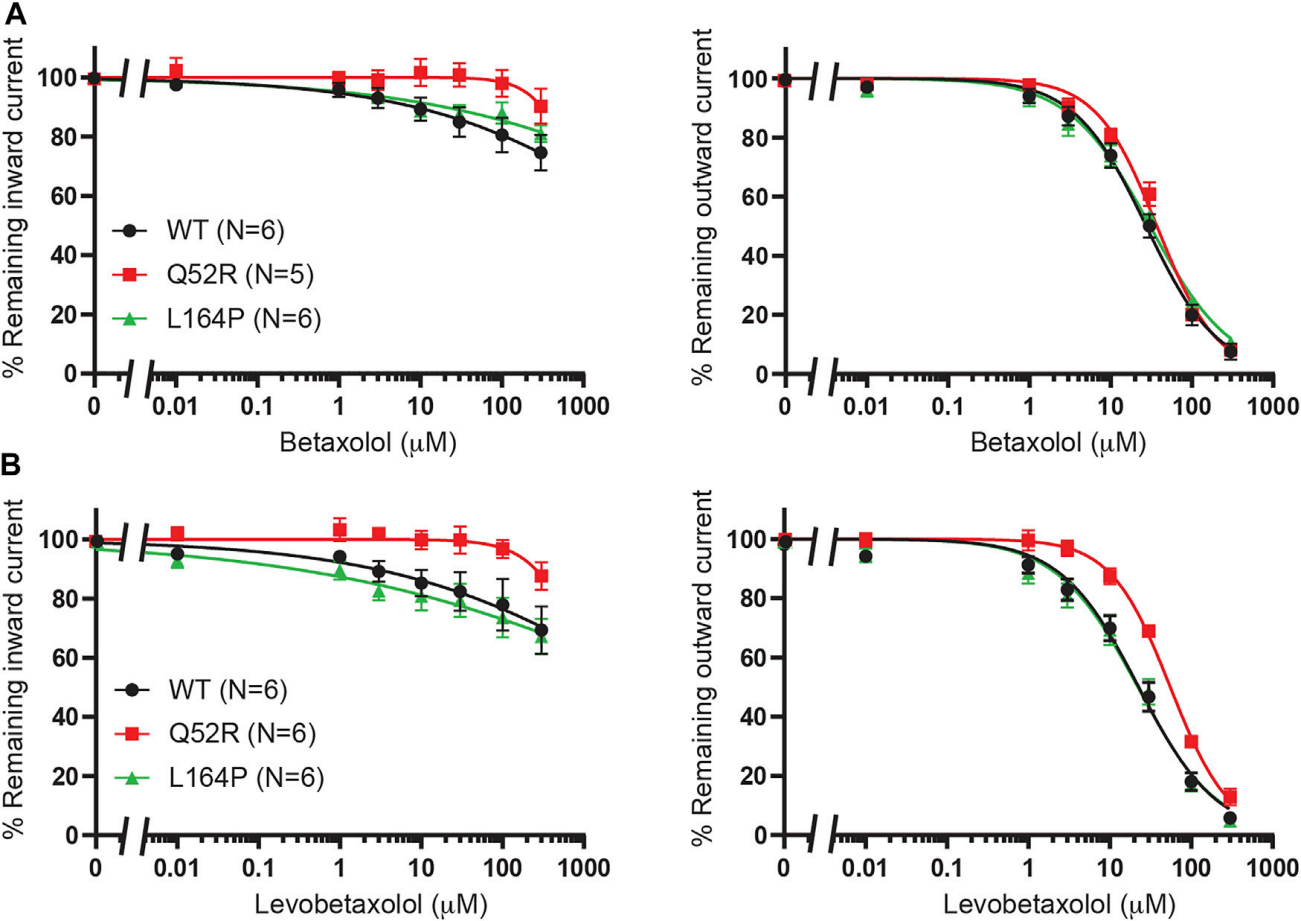
Inhibition of Q52R and L164P K_IR_6.2/SUR2a dependent current by betaxolol and levobetaxolol. IC_50_ curves of K_IR_6.2/SUR2a inward (at −80 mV), left panels, and outward (at +50 mV), right panels, current inhibition in response to increasing concentrations of **(A)** betaxolol and **(B)** levobetaxolol. Wildtype K_IR_6.2/SUR2a serves as control. Data were fitted with Hill equation to estimate the IC_50_ values. Data are shown as mean ± SEM. N depicts number of measurements on independent inside-out patches.

We previously demonstrated betaxolol mediated inhibition of K_IR_6.2/SUR2a current (Chen et al., 2019). To test the inhibitory capacity of betaxolol and its closely related homologue levobetaxolol (n^o^ 3 on Chen et al. (2019) hit-list) on Q52R and L164P mutants, we performed inside-out recordings on HEK293T cell membrane patches expressing either WT, Q52R or L164P K_IR_6.2/SUR2a channels. Both compounds did not significantly inhibit the inward component at −80 mV for concentrations up to 300 μM (Figures 3A,B). In contrast, outward current at +50 mV became inhibited by betaxolol with almost identical capacity for WT and mutants (IC_50_ 27.5 ± 2.1 μM, Hill coefficient −0.98 vs. 37.1 ± 2.1 μM, Hill coefficient −1.17 (n.s.) and 30.6 ± 2.3 μM, Hill coefficient −0.87 (n.s.), for WT, Q52R and L164P, respectively) (Figure 3A). Levobetaxolol inhibited the outward current of Q52R channels less potently than for WT and L164P channels (IC_50_ 22.7 ± 2.0 μM, Hill coefficient −0.91 vs. 55.3 ± 3.1 μM, Hill coefficient −1.19 (*p* < 0.01) and 21.8 ± 2.1 μM, Hill coefficient -0.88 (n.s.), for WT, Q52R and L164P, respectively) (Figure 3B). Interestingly, betaxolol also inhibited the classical inward rectifier K_IR_2.1 current with similar IC_50_ values (IC_50_ 29.8 ± 3.7 μM, Hill coefficient −0.79) (Supplementary Figure S2). Sequential mutation of the classical polyamine binding sites within the cytosolic (E224A, D259A, F254A, E299A) and transmembrane (D172A) pore domains resulted in a mild increase in IC_50_ values, except for E224A (approximately 35% inhibition at 100 μM) (Supplementary Figure S2).

Aiming to sample putative binding sites of betaxolol at reasonable computational costs, we carried out coarse-grained molecular dynamics (MD) simulations with the WT K_IR_6.2 channel pore in presence of ten betaxolol drug molecules randomly placed in the solvent. The reasons not to include the SUR subunit in these simulations were: 1) electrophysiology measurements show voltage dependent inhibition, 2) the Hill coefficient was around 1, and 3) our experimental data shows comparable inhibition of the different K_ATP_ mutants by betaxolol. These arguments indicate that the drug acts on the channel pore rather than on the SUR subunit.

**FIGURE 4:**
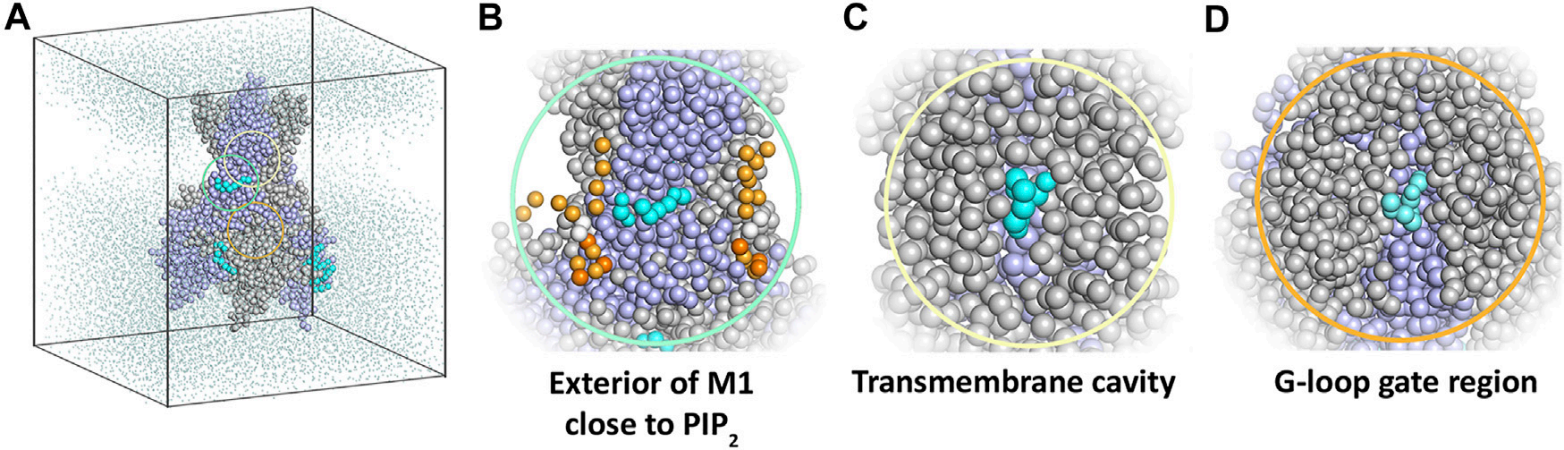
Coarse-grained MD simulation with the K_IR_6.2 channel pore and betaxolol. **(A)** Overview of a typical simulation system after 1 µs simulation. Different subunits of the K_IR_6.2 channel are shown as grey and lilac spheres, respectively. Bound betaxolol molecules are shown as cyan spheres. Water molecules are illustrated as dots, while lipid molecules are omitted for clarity. **(B)** Close-up of the exterior of M1, where a betaxolol molecule is bound near the PIP_2_ binding site. PIP_2_ molecules are shown as orange spheres. **(C)** Close-up of the transmembrane cavity. **(D)** Close-up of the G-loop region.

Ten replicate runs of 1 µs-long MD simulations unraveled three main binding sites for the drug, which are located close to the PIP_2_ binding site at the surface of helix M1, in the transmembrane cavity, and in the cytoplasmic domain at the G-loop region (Figure 4).

**FIGURE 5:**
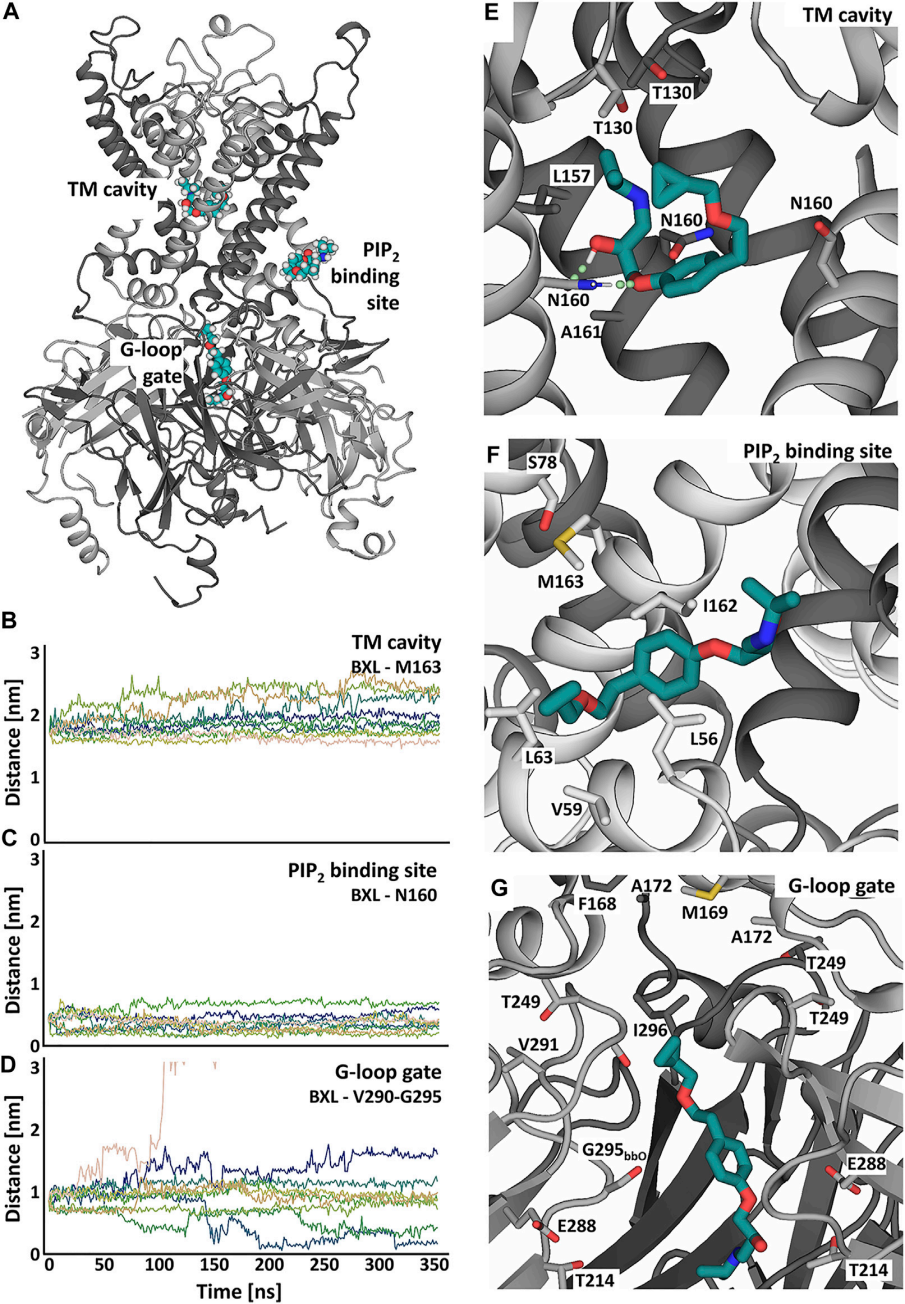
Putative betaxolol binding sites and stability investigated in atomistic MD simulations with the K_IR_6.2 channel pore. **(A)** Overview of the binding sites of betaxolol to the K_IR_ channel pore. Different K_IR_ subunits are shown in cartoon representation and colored in dark and light grey. The different betaxolol molecules are shown in teal. **(B–D)** Distances between betaxolol and the K_IR_6.2 channel in 9 replica of 350ns-long MD simulations. The differently colored lines represent the distances of the docked betaxolol molecules to the respective binding sites in the different runs. **(E–G)** Close-ups of the betaxolol binding sites at TM-cavity **(E)**, PIP_2_ binding site **(F)**, G-loop gate **(G)** seen at the end of run1 of the all-atom MD simulations. The drug is shown as teal sticks, with hydrogen bonds shown as green dotted lines.

In order to analyze the binding sites in more detail, we performed atomistic MD simulations of the K_IR_6.2 channel pore in the presence of betaxolol docked at the identified interaction sites Figure 5A (Supplementary Table S1). Figures 5E–G shows typical snapshots of the respective betaxolol binding poses observed in the atomistic MD simulations. The binding site near PIP_2_ is formed by a cleft between the lower halves of transmembrane helix 1 (TM1) and 2 (TM2) of one subunit. Betaxolol predominantly engages in hydrophobic interactions with residues L56, L63 and M163. This binding pose is reminiscent of the binding site of rosiglitazone to the K_IR_6.1 channel pore identified in a previous study (Chen et al., 2019).

Binding of betaxolol in the transmembrane cavity is mediated by hydrogen bonds to N160 (Figure 5E, Supplementary Figure S3B), which can be observed in approximately 70% of the simulation time, as well as hydrophobic interactions to L157, A161 and T130. The third binding site is located in the cytoplasmic domain near the G-loop gate, which constitutes another important regulative barrier for ion conduction. Betaxolol forms hydrophobic interactions with residues V290, I296, A172, M169 and the HBC gate composing residue F168. Additional hydrogen bonds can be observed between the drug and T214, E288 and the backbone oxygens of S212 and G295 (Supplementary Figures S3C–F).

Figures 5B–D shows distance measurements of the docked betaxolol molecules to the respective binding sites observed in 9 runs of 350 ns long. The analysis reveals a stable drug interaction near the PIP_2_ binding site and the TM cavity (Figures 5B,C), since dissociation of betaxolol is not observed in any of the simulations. For the interaction near the G-loop gate, a greater deviation of the drug from its original binding pose can be observed. Figure 5D shows that the distance between betaxolol and the G-loop gate composing residues V290-G295 decreases in two runs, which is caused by betaxolol detaching from its initial binding site and moving towards the narrowest parts of the G-loop gate, where it remains stable for the rest of the simulation time. Similar observations could be made in the two additional 1 μs-long MD simulations (Supplementary Figure S4).

Taken together, our simulations suggest that betaxolol plugs the conduction pathway by interacting with pore lining residues. This also provides an explanation for its voltage dependent block (outward >> inward) observed in electrophysiology measurements. In addition to this pore block, betaxolol binding near the PIP_2_ binding site might also influence the conductivity, a phenomenon which has already been described for other K_IR_ channel blockers (López-Izquierdo et al., 2011; Ponce-Balbuena et al., 2012; Koepple et al., 2017; Scherer et al., 2017).

### Travoprost, Latanoprost and Dinoprostone

Given the relatively low potency (mid-micromolar) and state dependency of betaxolol and levobetaxolol, we also tested more lipophilic compounds with higher affinity towards K_IR_6.2/SUR2a channels. Travoprost, a synthetic prostaglandin derivative, was identified as a drug that inhibits both outward and inward currents (Chen et al., 2019). In addition, we decided to test a naturally occurring prostaglandin [dinoprostone, n^o^ 4 on Chen et al. (2019) hit-list] and an additional synthetic prostaglanding (latanoprost).

**FIGURE 6:**
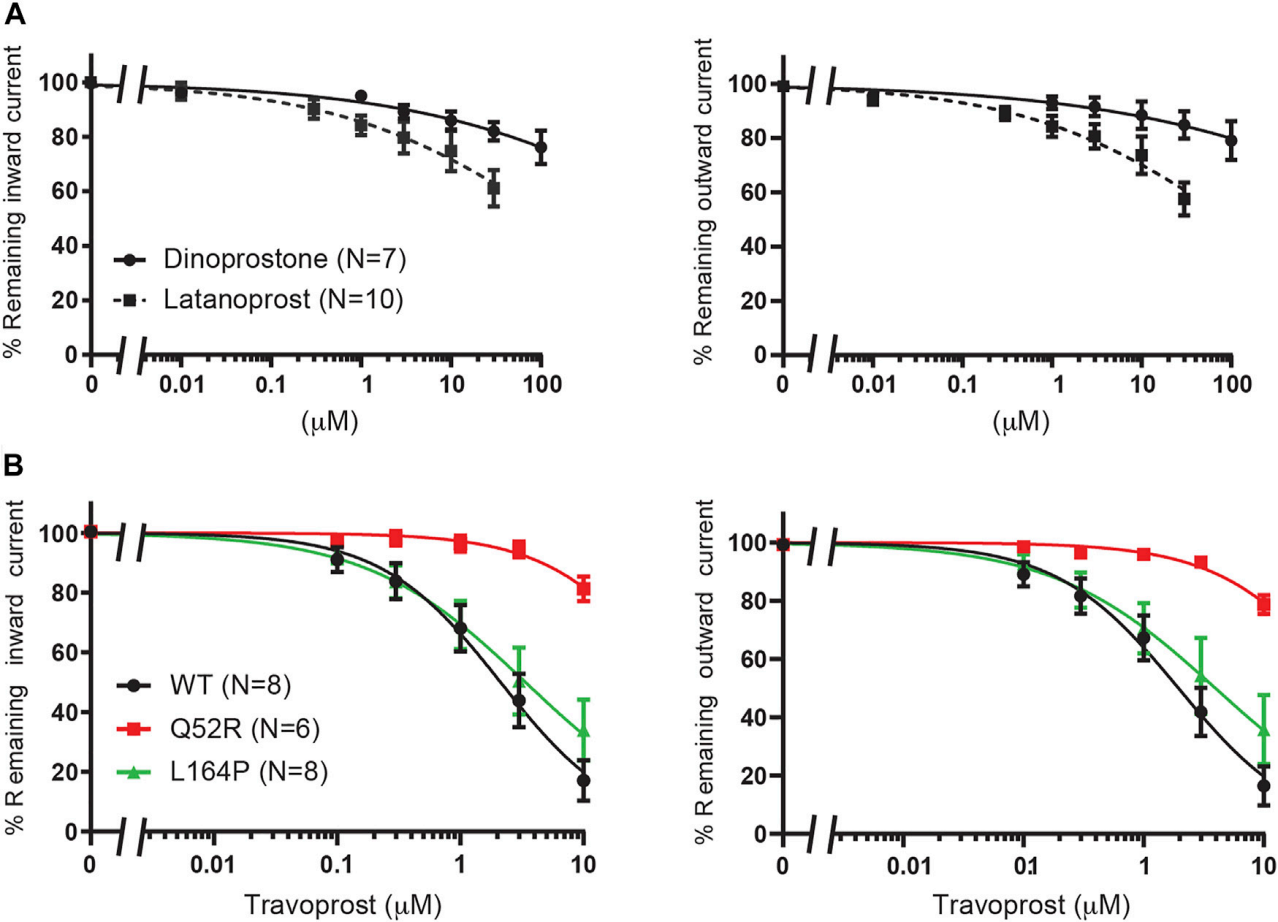
Evaluation of K_IR_6.2/SUR2a dependent currents in response to dinoprostone, latanoprost and travoprost. **(A)** IC_50_ curves of WT K_IR_6.2/SUR2a inward (at −80 mV), left panel, and outward (at +50 mV), right panel, current inhibition in response to increasing concentrations of dinoprostone or latanoprost. **(B)** IC_50_ curves of WT, Q52R and L164P K_IR_6.2/SUR2a inward (at −80 mV), left panel, and outward (at +50 mV), right panel, current inhibition in response to increasing concentrations of travoprost. Data were fitted with Hill equation to estimate the IC_50_ values. Data are shown as mean ± SEM. N depicts number of measurements on independent inside-out patches.

In order to investigate the reputative binding sites of travoprost, latanoprost and dinoprostone, we compared their inhibiting capacity on WT K_IR_6.2/SUR2a channel excised membrane patches, similar as described in *Betaxolol and Levobetaxolol*. Whereas, travoprost mediated potent inhibition (see below), considerably less inhibition was observed with latanoprost (approximately 35–40% inhibition at 30 μM), and no inhibition was observed with dinoprostone (approximately 10–20% inhibition at 100 μM) (Figure 6A). Subsequently, travoprost was tested on WT, Q52R and L164P channel patches (Figure 6B). Both inward and outward currents of WT channel were inhibited (IC_50_ 2.1 ± 0.4 μM, Hill coefficient −0.90 and 2.0 ± 0.3 μM, Hill coefficient −0.86, respectively), while inhibition of L164P channels showed similar IC_50_ values, (IC_50_ 3.4 ± 0.8 μM, Hill coefficient −0.67 (n.s.) and 4.0 ± 1.2 μM, Hill coefficient −0.64 (n.s.) for inward and outward L164P, respectively). No significant inhibition of Q52R channels was observed (approximately 15–20% inhibition at 10 μM), We next carried out coarse-grained molecular dynamics (MD) simulations with travoprost and latanoprost to identify their putative binding sites. In contrast to betaxolol, the experimental data for these two drugs does not allow the exclusion of binding sites on SUR. Hence, the coarse-grained MD simulations were conducted with the whole K_ATP_ channel complex, including the four SUR subunits. As a starting point for the simulations, we chose the highest resolution Cryo-EM structure pair available [PBD: 6C3O/6C3P (Lee et al., 2017)], which shows two very different orientations of the SUR subunits with regard to the K_IR_6.2 channel pore. For both Cryo-EM structures, a total of 30 μs coarse-grained MD simulations were conducted in the presence of travoprost and latanoprost, respectively (Supplementary Table S1).

**FIGURE 7:**
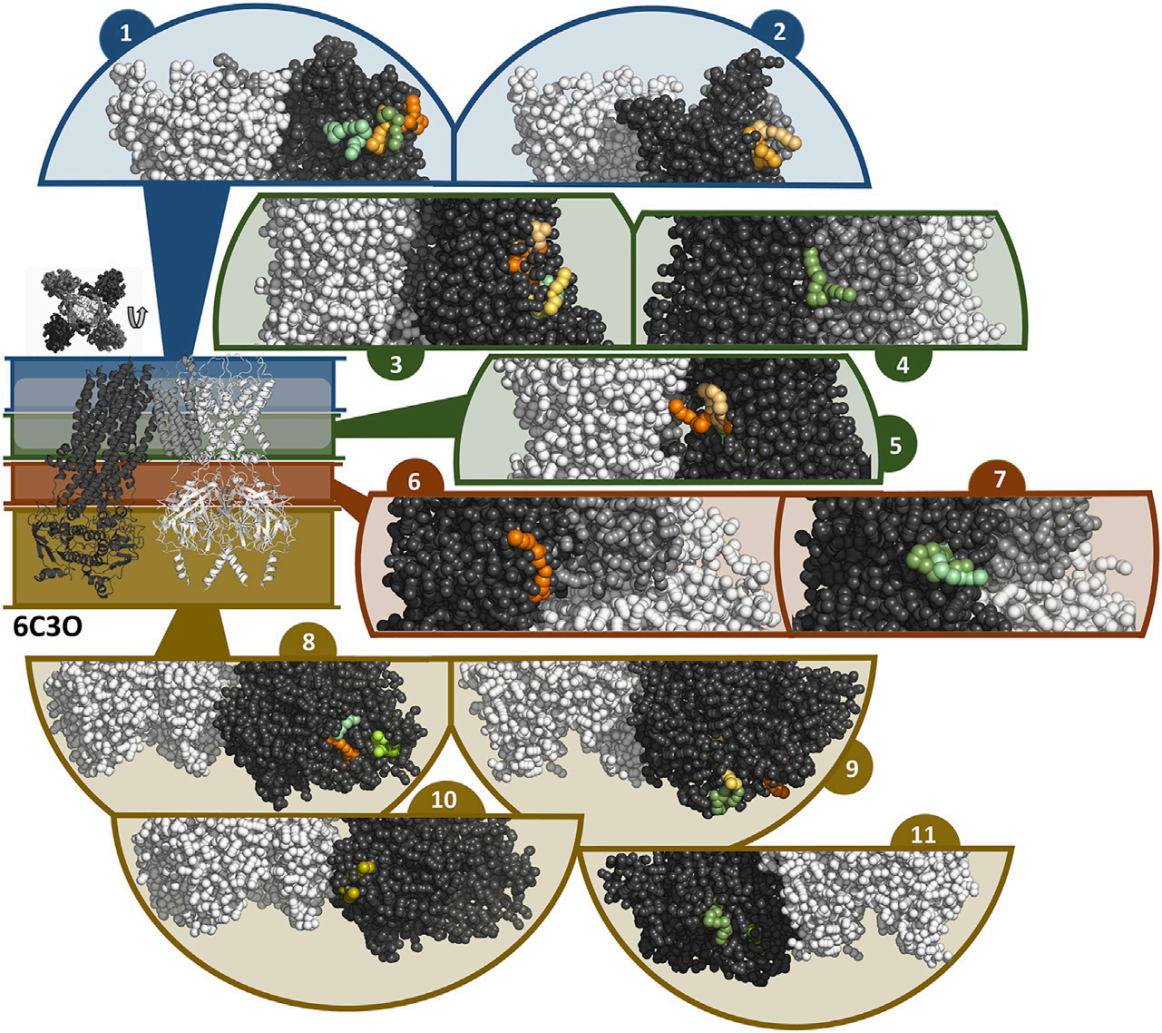
Binding sites of travoprost and latanoprost found in a total of 60 μs coarse-grained MD simulations with the 6C3O structure. The smaller figure on the left shows the overall structure of the 6C3O channel, the location of the lipid bilayer membrane is indicated with a white bar. For the sake of clarity, the structure is divided in different zones, which are shown in different colors (blue = extracellular part as well as the outer lipid layer of membrane, green = inner lipid layer of membrane, red = cytoplasmic domain near cell membrane, yellow = cytoplasmic domain). The figure above the overall structure shows the orientation of the SUR subunits in a top-down view of the channel. The different sub figures show close-ups of different interaction sites of the drugs to the channel. Travoprost is shown in shades of green, latanoprost in shades of orange and yellow. In each of the close-ups, all four subunits of the K_IR_ channel are shown colored in white. For SUR, only one subunit is shown. The TM0 of SUR colored in middle grey, while the rest of SUR is colored in dark grey.

**FIGURE 8:**
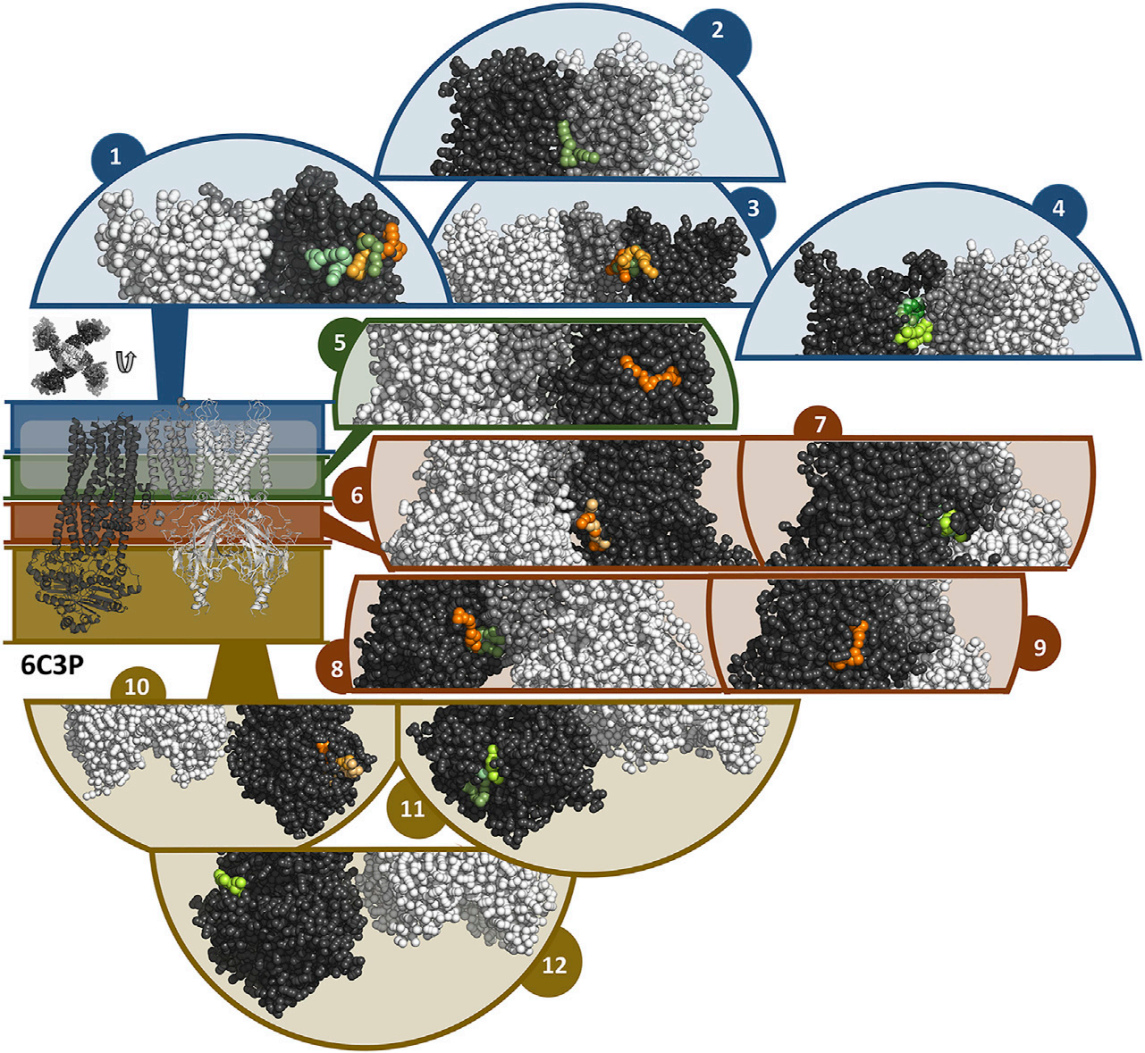
Binding sites of travoprost and latanoprost found in a total of 60 μs coarse-grained MD simulations with the 6C3P structure. The smaller figure on the left shows the overall structure of the 6C3P channel, the location of the lipid bilayer membrane is indicated with a white bar. For the sake of clarity, the structure is divided in different zones, which are shown in different colors (blue = extracellular part as well as the outer lipid layer of membrane, green = inner lipid layer of membrane, red = cytoplasmic domain near cell membrane, yellow = cytoplasmic domain). The figure above the overall structure shows the orientation of the SUR subunits in a top-down view of the channel. The different sub figures show close-ups of different interaction sites of the drugs to the channel. Travoprost is shown in shades of green, latanoprost in shades of orange and yellow. In each of the close-ups, all four subunits of the K_IR_ channel are shown colored in white. For SUR, only one subunit is shown. The TM0 of SUR colored in middle grey, while the rest of SUR is colored in dark grey.

In general, the coarse-grained MD simulations unraveled a strong affinity of travoprost and latanoprost to the SUR subunits, where a broad range of binding sites could be identified. All of the drugs in the simulation systems bind to the protein complex (Supplementary Figure S5), whereby the interaction sites are almost exclusively located on SUR. A summary of the binding sites found in the simulations with the 6C3O and 6C3P structures is shown in Figures 7, 8, respectively. Since most of the binding sites were observed more than once, either by being detected in different simulations or in the same simulations on different subunits, similar binding poses were collected in interaction clusters in order to give a comprehensive overview of the affinities.

In the simulations with 6C3O and the 6C3P, 11 and 12 interaction clusters can be identified, respectively. Importantly, clusters 1, 4, 5, and 10 are identical between the structures and hence are detected independently of the starting configuration of the simulation. Cluster 1 is located between the POPC membrane and TMD1 as well as TMD2, whereby the observed binding poses deviate from each other to a greater extent than in other clusters. This site is quite frequently occupied, as it is found by 2 travoprost molecules and 3 latanoprost molecules, as well as 1 travoprost and 4 latanoprost molecules in simulations with 6C3O and 6C3P, respectively. Clusters 4 and 5 are located approximately at the level of the intracellular boundary of the membrane. Cluster 4 is located at the interface of TMD0 and TMD1 and is only occupied by travoprost molecules (1 in 6C3O, 4 in 6C3P), while both travoprost and latanoprost are seen in cluster 5 at the surface of TMD2 (1 travoprost and 2 latanoprosts molecules in 6C3O, 1 latanoprost molecule in 6C3P). In simulations with the 6C3O structure, the latter interaction site is framed by TMD2 and the K_IR_ channel, whereas the more extended, “propeller”-like conformation of 6C3P places this binding site between SUR and the membrane. Another interaction site observed for both 6C3O and 6C3P is cluster 10, which is located at the cytoplasmic part of SUR on the TMD2.

The rest of the interaction clusters are only seen for either 6C3O or 6C3P. For 6C3O, interaction cluster 3 is well occupied (2 travoprost molecules, 3 latanoprost molecules) and contains a range of different binding poses at the surface of TMD1. Another interaction, shown in cluster 2, is located at TMD1 approximately at the same level as cluster 1 and is only observed for latanoprost. The binding pose shown in cluster 6 is found by one travoprost molecule and is in direct vicinity of cluster 4, while interaction cluster 7 is located below the well-populated cluster 3 at the surface of TMD2. Furthermore, a variety of affinity sites (clusters 8, 9 and 11) were identified at the surface of the cytoplasmic domain.

In the simulations with 6C3P, interaction cluster 2 stands out since it is the only cluster in direct contact with the K_IR_6.2 channel pore. This site is occupied by one travoprost molecule in all simulations with 6C3P and is sandwiched between the membrane and the K_ATP_ channel, where it makes contact with both the TM2 of K_IR_ and the TMD0 of SUR. Cluster 3 is approximately on the same level as cluster 4, interacting with TMD1 in vicinity of TMD0. The interaction cluster 8 is positioned at the surface of TMD0 and TMD1 near the cytoplasmic boundary of the membrane, and separated by the lower ends of these two interacting helices. Cluster 6 is close to the ATP binding site on the K_IR_ subunit. Two latanoprost as well as one travoprost molecule and one latanoprost molecule are occupying the interaction sites in cluster 6 and 8, respectively. Cluster 9 is below cluster 3 found in the simulations with 6C3O, but is only detected by one travoprost molecule, whereas two additional binding poses are seen at the lower cytoplasmic end of SUR, represented as clusters 11 and 12. Interestingly, cluster 11 is close to the ATP binding site at the degenerate nucleotide binding domain of the SUR subunit (cite Lee et al., 2017). Further information about the number of drug molecules in each interaction cluster as well as distance measurements and contacting residues are summarized in Supplementary Table S3.

### Fluvastatin, Ethosuximide and Pazopanib

**FIGURE 9:**
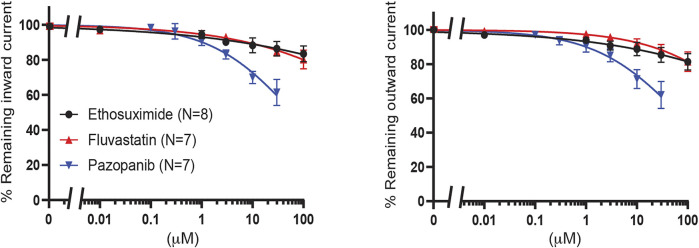
Evaluation of K_IR_6.2/SUR2a dependent currents in response to ethosuximide, fluvastatin and pazopanib. IC_50_ curves of WT K_IR_6.2/SUR2a inward (at −80 mV), left panel, and outward (at +50 mV), right panel, current inhibition in response to increasing concentrations drugs. Data were fitted with Hill equation to estimate the IC_50_ values. Data are shown as mean ± SEM. N depicts number of measurements on independent inside-out patches.

Finally, fluvastatin [n^o^ 2 on Chen et al. (2019) hit list], ethosuximide and pazopanib were tested. Ethosuximide is a K_IR_2.1 and K_IR_3 inhibitor with favorable properties for crossing the blood brain barrier (Colombo et al., 2008; Kobayashi et al., 2009; Huang and Kuo 2015). Clinically, pazopanib induces hypoglycemia as an adverse effect in 0.1–1% of patients. All three compounds did not significantly inhibit K_IR_6.2/SUR2a current (approximately 20% inhibition for fluvastatin and ethosuximide at 100 μM, and approximately 40% inhibition at 30 μM for pazopanib) (Figure 9).

## Discussion

In this work, we use a combination of electrophysiology and molecular modelling in order to investigate putative binding sites of betaxolol, travoprost and latanoprost as well as the molecular mechanism underlying K_ATP_ channel inhibition by these compounds. Inside-out recordings presented here were performed in the absence of Mg-ATP and ADP in the bath solution, to best mimic the MD conditions. This most likely resulted in 6–11 fold higher glibenclamide IC_50_ values than in the presence of 0.15 mM MgATP (Houtman et al., 2019), as seen also in measurements of tolbutamide block in presence and absence of ATP (Miyamura et al., 2000). Nevertheless, Q52R and L164P displayed reduced glibenclamide sensitivity as expected, which therefore is a MgATP independent characteristic. This latter finding also applies to the reduced travoprost mediated inhibition sensitivity as observed for the Q52R mutant.

We established the effects of each compound on both the inward and outward component of the I_KIR6.2/SUR2a_ current. From a physiological perspective, the outward component is most relevant, but when comparing the voltage-dependence of block (inward vs. outward) insights can be gained on the nature of block, e.g., direct pore block vs. inference in PIP_2_-channel interaction.

### Betaxolol

MD simulations with betaxolol were carried out in presence of the K_IR_6.2 channel pore without SUR1. The motivation for this setup is twofold: firstly, electrophysiological experiments show that betaxolol inhibits the WT as well as many of the tested mutations, including L164P and Q52R, in a similar fashion (Figure 3A). Secondly, the mutations L164P and Q52R were reported to impair the coupling of the K_IR_ channel to SUR (Tammaro et al., 2008; Tammaro et al., 2006, Pratt et al., 2012) and to be partially resistant to inhibition by SUR-binding sulfonylureas (Pearson, et al., 2006; Tammaro et al., 2008). Since an allosteric inhibition would require drug binding to overcome the disruptive effect of both mutations in a similar fashion, we deem it likely that betaxolol blocks the open channel pore instead of inhibiting the channel allosterically. In addition, the Hill coefficient of -0.98 for WT channels is in line with pore block. Assuming a pore block, it is sensible to simulate the K_ATP_ complex without the SUR subunits, since this reduction of the system size drastically decreases the computational cost of simulating the system.

Coarse-grained and atomistic MD simulations show binding of betaxolol at the transmembrane cavity and near the G-loop gate, where it plugs the ion conduction pathway of the K_ATP_ channel (Figures 4C,D, 5E,G). A resembling blockage can be commonly encountered in the K_IR_2.x channel family, where a range of compounds, including pentamidine (De Boer et al., 2010), quinidine (Koepple et al., 2017) and chloroquine (Rodriguez-Menchaca et al., 2008), have been shown to prevent ion flux by blocking the cytoplasmic pore.

The betaxolol binding sites at the transmembrane cavity and near the G-loop gate are situated in vicinity to the disease mutation L164P and could generally explain why betaxolol effectively inhibits this mutant. In a previous study, we predicted that the inserted proline mainly leads to a widening of the pore and increased solvation of the gates, thereby preventing proper closure of the channel (Bründl et al., 2021). Thus, binding of the drug to the inner cavity and/or the G-loop gate would prevent ion flux by blocking the permeation path through the channel.

The disease mutation Q52R is located in the loop connecting the slide helix with the C-terminal domain (CTD) of the of K_IR_ channel, in vicinity to the PIP_2_ binding site (Puljung, 2018) (Figure 1A). Furthermore, the 6C3P structure shows that Q52 extends towards the cytoplasmic loop L0 of SUR1, where it resides within 5 Å of residue E203 (Figure 1A). The role of the interaction between Q52- K_IR_6.2 and E203-SUR1 in mediating K_IR_6.2 gating by SUR was confirmed by a mutation study, which demonstrated that the crosslinking of E203C in SUR1 and Q52C in K_IR_6.2 *via* a Cys-Cys bridge locks the channel in a closed state (Pratt et al., 2012). However, the effect of the Q52R mutation on the channel structure is currently unclear and thus, needs to be elucidated in future investigations. Nevertheless, putative pore blockers such as betaxolol and levobetaxolol can inhibit this mutant as well, and therefore might provide a basis for drug development for sulfonyl-urea resistant DEND mutations.

Nowadays, the most successful therapy for the DEND syndrome is based on K_ATP_ channel inhibition by sulfonylureas, which bind to the SUR subunits and exert inhibition in an allosteric manner. The inability of sulfonylureas to cross the blood-brain-barrier and alleviate the neurological problems of the disease, as well as the fact that some mutations are resistant to these compounds call for the development of a more diverse set of K_ATP_ inhibitors (Kharade et al., 2016). Betaxolol is different from the sulfonylureas in that it blocks the ion conduction pathway, and thus can be counted among a small set of K_ATP_ channel inhibitors targeting the channel pore. Other compounds with an affinity to the K_IR_ channel are verapamil (Ninomiya et al., 2003), tamoxifen (Ponce-Balbuena et al., 2010) and rosiglitazone (Yu et al., 2012), whereby the latter two have been reported to interfere with the channel activation by PIP_2_. In addition to the development of chemically based inward rectifier channel inhibitors and activators (Van der Schoor et al., 2020; Walsh, 2020), progress is made also in the field of short peptide toxins as channel inhibitors (Doupnik, 2017). The small protein SpTx-1 was shown to inhibit a range of permanent ND causing mutations, including Q52R, by binding to K_IR_6.2 (Ramu et al., 2018). Furthermore, the recently studied centipede toxin, SsTx-4, displayed low nanomolar IC_50_ values for K_IR_6.2/SUR1 channels, including the Q52R mutant channel (Tang et al., 2021).

Upon oral dosing (40 mg, 15 days p.o.), betaxolol plasmalevels of approximately 0.54 μM were found (Lipworth et al., 1991). Whereas IC_50_ values around 30 μM were observed for K_IR_6.2/SUR2a outward current, approximately 20% reduction of outward current is already found at 5 μM. Drugs with an affinity to the K_IR_6.2 channel pore might therefore provide a powerful tool to inhibit sulfonylurea-resistant DEND mutations, which makes betaxolol a promising starting point for the development of a novel medication against this syndrome.

However, it should be mentioned that betaxolol is not able to cross the blood-brain-barrier (BBB) to a meaningful extent and thus, is unlikely to suppress the neurological and developmental symptoms associated with the DEND syndrome.

In addition to pore blockage, the simulations unraveled another interaction site for betaxolol, which is located near the binding site of PIP_2_. In support of this finding, certain drugs were reported affecting K_IR_ channels by blocking the pore and simultaneously exerting an allosteric inhibition by interacting with to the PIP_2_ binding site (López-Izquierdo et al., 2011; Koepple et al., 2017; Scherer et al., 2017). One of these compounds is chloroquine, which has been shown to inhibit K_IR_6.2 containing K_ATP_ channels in this two-fold manner (Ponce-Balbuena et al., 2012). A fast voltage-dependent inhibition observed at positive potentials was suggested to originate in a blockage of the cytoplasmic channel pore, while a slower inhibition might stem from an impaired channel activation by PIP_2_ caused by drug interference with this site. The experimentally observed voltage dependence of K_ATP_ inhibition by betaxolol (Chen et al., 2019, and current data) could be explained by a channel block in the central cavity region or close to the G-loop, and therefore supports the hypothesis of betaxolol being a K_ATP_ pore blocker. However, drug binding near the PIP_2_ binding site might additionally influence the conductivity, as it is seen for chloroquine, and we therefore cannot exclude an allosteric effect involved in K_ATP_ channel inhibition by betaxolol.

### Prostaglandins

Travoprost and latanoprost are both synthetic analogues of prostaglandin, which belongs to the group of eicosanoids. In coarse-grained MD simulations with the K_ATP_ channel, travoprost and latanoprost exhibited a high affinity to the SUR subunits. Our functional measurements demonstrate that travoprost, and to a lesser extent latanoprost, inhibits both inward and outward components of the WT I_KATP_ channels, in contrast to betaxolol which displays voltage dependent block (outward >> inward). Based on these observations, we consider it more likely that travoprost and latanoprost act by binding to SUR1 rather than to the K_IR_6.2 channel pore. The variety of interactions observed for both drugs could be interpreted as the presence of multiple binding sites on the SUR subunits. Although the data obtained from the MD simulations indicates the presence of multiple prostaglandin interaction sites with similar affinity, we cannot exclude the existence of one or more high affinity binding sites.

Interestingly, in the simulations of 6C3P, we found two binding clusters near sites important for K_ATP_ regulation. Cluster 6 shows binding of travoprost and latanoprost in proximity to the inhibitory ATP binding site at the K_IR_ subunit. Cluster 11 shows binding of travoprost to the degenerate site of the SUR subunit. Since ATP binding to either K_IR_ or SUR exhibits regulatory effects, potential binding of compounds to or close to these binding sites might compete with or change the ability of ATP regulation of this channel. However, since the details of K_ATP_ regulation are still unknown, the mechanism by which these compounds modulate the channel remains obscure.

In the context of the DEND syndrome, a therapeutic approach based on a drug with binding affinity to SUR might not be the best choice. The reason for this is that some of the DEND syndrome causing mutations, including Q52R and L164P, are known to impair the coupling of SUR to the K_IR_ channel pore (Tammaro et al., 2006; Tammaro et al., 2008, Pratt et al., 2012). This could undermine the effect of a SUR-bound K_ATP_ channel inhibitor and adds an unwanted factor of uncertainty in predicting the efficiency of the inhibition. A further characterization of the binding behavior of this drug class to K_ATP_ channels might be interesting for the treatment of other K_ATP_ channelopathies.

### Additional Tested Compounds

Fluvastatin is on the Chen hitlist (n^o^ 2), but did not result in K_IR_6.2/SUR2a inhibition. Pharmacophore based database searches provides enrichment of hit rates compared to random sampling, but does comes with false positives (Seidel et al., 2019), like in this case fluvastatin.

A 2010 case report described improved glycemic control in a prostate cancer patient, who also presented glibenclamide resistant Type 2 diabetes, upon treatment with pazopanib (400 mg/p.d.) (Böhm et al., 2010). The authors suggested that pazopanib mediated inhibition of PDGF-receptor signaling is the key factor in this finding. Our result of a non-significant change in I_KIR6.2/SUR2a_ upon application of 30 μM pazopanib does not disagree with their suggestion. However, at a daily dosing of 800 mg, peak plasma concentrations were found as high as 132 μM (NIH Daily Med 2014). We did not perform functional measurements at such concentrations.

### Limitations

Our study comes with several limitations. A shortcoming of our study is that we exclusively simulate channels with a closed pore module due to a lack of open K_ATP_ structures. Two studies, which modelled the opening of K_IR_2 channels either by forcing the channel into a conductive state (Li et al., 2015) or by introducing channel-opening mutations (Zangerl-Plessl et al., 2020), identified motions in the CTD, bending of the inner transmembrane helix (TM2), and a widening at the Helix Bundle Crossing (HBC) gate to be associated with the acquisition of an activated state. However, a rearrangement of the residues lining the pore of the channel was not reported. Based on these findings, one can expect that the residues in the TM cavity remain available for drug interaction independently of the conductive state of the channel and thus, the betaxolol block at this site to be relatively unchanged upon channel opening. Nevertheless, channel opening was accompanied by structural changes at the G-loop region in targeted MD simulations (Li et al., 2015). Hence, the possibility of an altered binding behavior of betaxolol near the G-loop gate in the open channel should not be neglected. A further limitation to the closed crystal structure is that betaxolol is not able to interact with the HBC gate, and we therefore cannot assess the interplay of the compound with the gate comprising residue F168 in the simulations. Secondly, a number of symptoms, including muscle weakness, of the DEND syndrome is generally considered to involve the K_IR_6.2/SUR1 channels (Clark et al., 2010). Our experimental evaluation uses K_IR_6.2/SUR2a channels instead. It was shown that the SUR isoform is important to confer the cellular metabolic status to I_KATP_ activity of a mutant channel (Clark et al., 2010). In our inside-out measurements we did not took the metabolic status into account, as Mg-ATP and ADP were not included in the bath solution. Therefore, we reason that the SUR isoform is not essential in our current experimental evaluations. Moreover, changes in Q52R single channel kinetics are independent of SUR isoform (Tammaro et al., 2006). Finally, we did not confirm potential binding sites as suggested by MD by mutational analysis in the current study, which could be considered as a limitation.

## Data Availability

The original contributions presented in the study are included in the article/Supplementary Material, further inquiries can be directed to the corresponding author.
