# Supplementary material for: Development of IKATP Ion Channel Blockers Targeting Sulfonylurea Resistant Mutant KIR6.2 Based Channels for Treating DEND Syndrome
Source: Front Pharmacol. 2022 Jan 14;12:814066. doi: 10.3389/fphar.2021.814066 (PMC8795863; doi:10.3389/fphar.2021.814066)
Supplement: Supplementary file 1 [file DataSheet1.pdf]

Supplementary Material

1 Supplementary Figures and Tables

1.1 Supplementary Figures

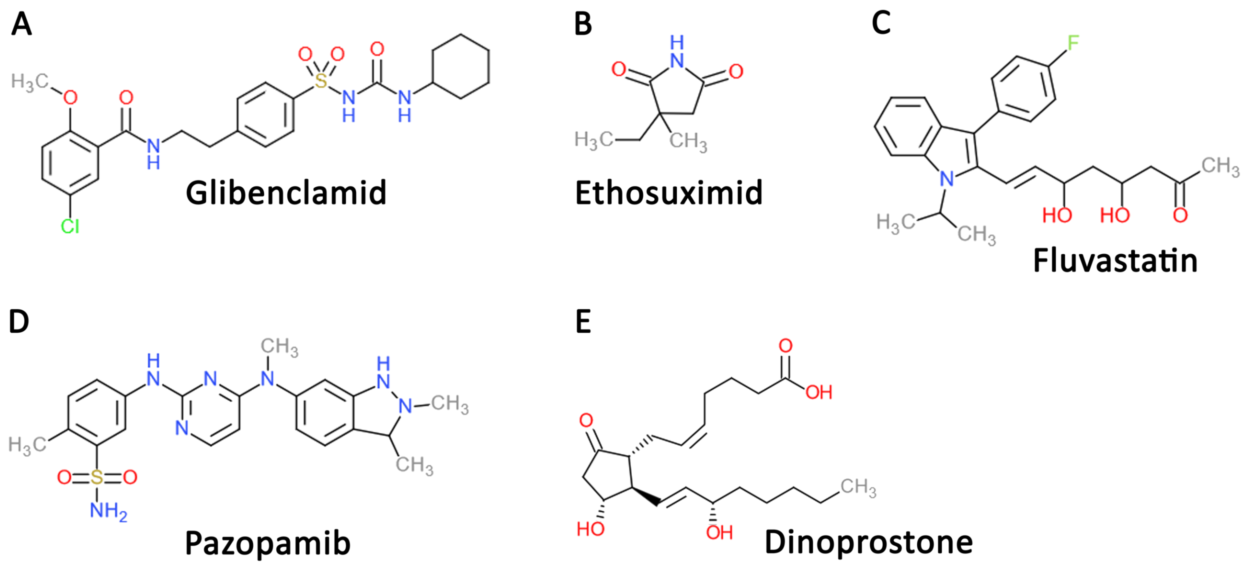

Supplementary Figure S1. (**A**) Chemical structure of glibenclamide. (**B**) Chemical structure of ethosuximide. (**C**) Chemical structure of fluvastatin. (**D**) Chemical structure of pazopamib. (**E**) Chemical structure of dinoprostone.

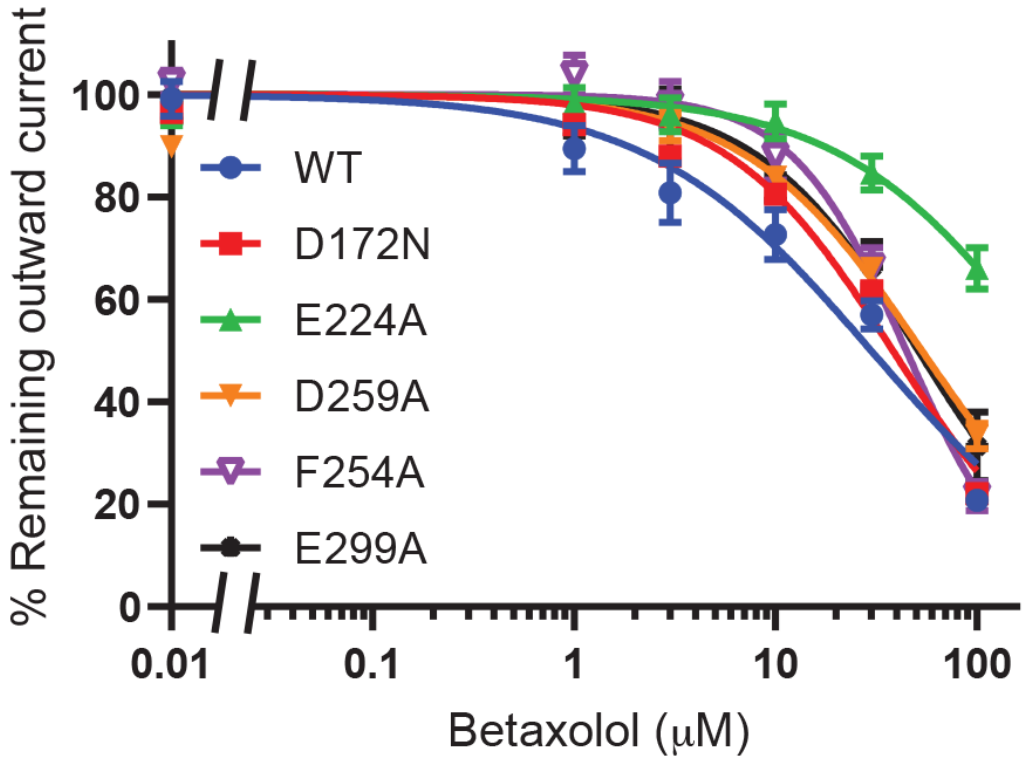

Supplementary Figure S2. Outward $I_{KIR2.1}$ from HEK293T cells transiently transfected with WT and cytosolic (E224A, D259A, F254A and E299A) and transmembrane (D172N) pore mutants exposed to increasing concentrations of betaxolol.

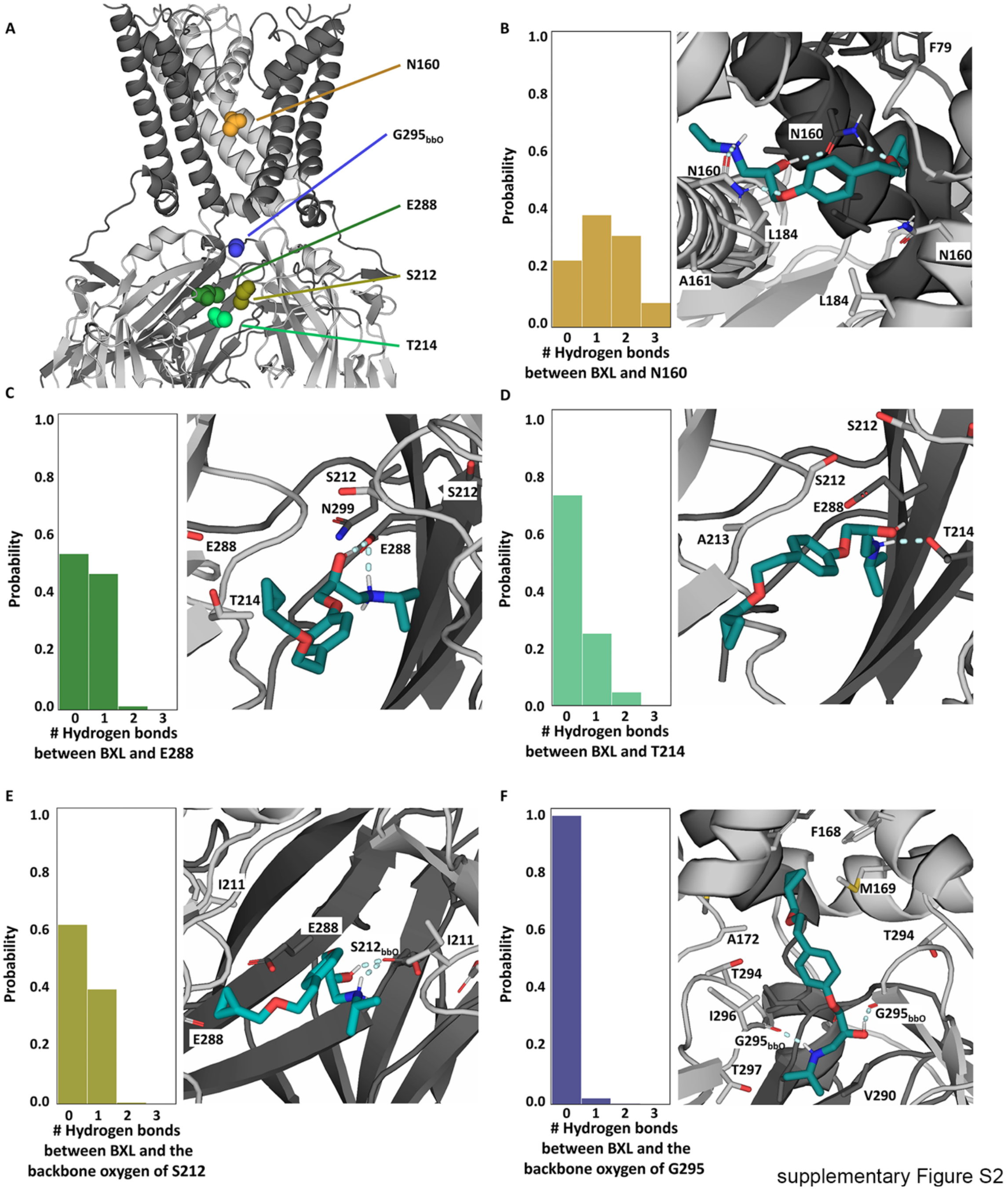

Supplementary Figure S3. Hydrogen bonds between betaxolol and K_{IR} pore residues observed in the atomistic MD simulations. (**A**) Three K_{IR} channel subunits are shown in different shades of grey. The residues, which are observed to form hydrogen bonds to betaxolol are shown in different colors. (**B-F**) Left: Histogram of the number of hydrogen bonds between betaxolol and N160 (**B**)), the backbone oxygen of S212 (**C**)), T214 (**D**)), E288 (**E**)) and the backbone oxygen of G295 (**F**)) observed in a total of 5.15 µs atomistic MD simulations. Right: Close-up of the hydrogen bonds between betaxolol and the respective residues. Betaxolol is shown as teal sticks and the hydrogen bond as light blue dashed lines.

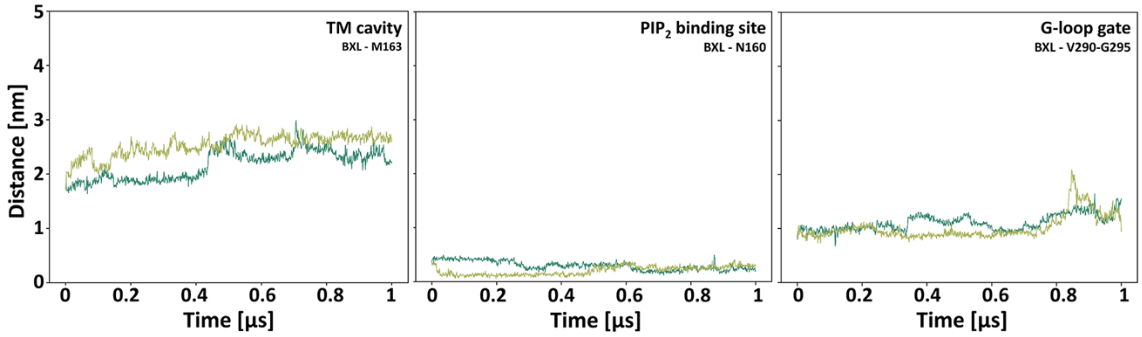

Supplementary Figure S4. Distances between betaxolol and the KIR6.2 channel pore based on 2 replica of 1µs-long all-atom MD simulations. The differently colored lines represent the distances of the docked betaxolol molecules to the respective binding sites in the different runs.

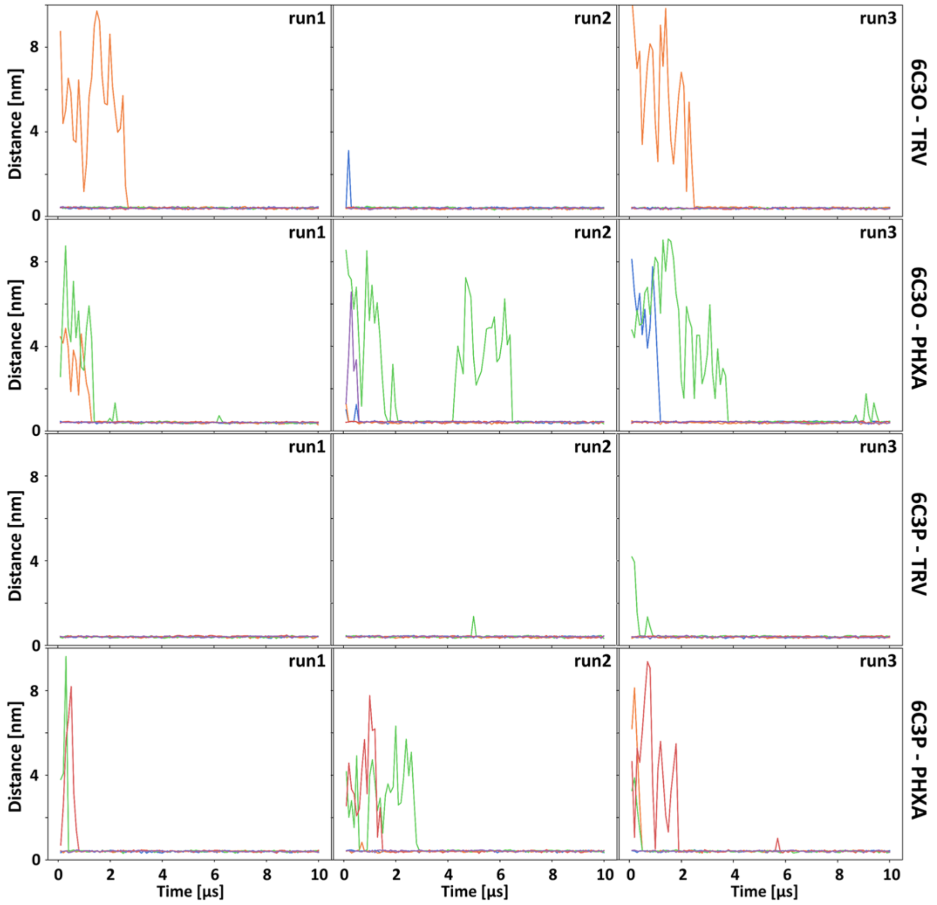

Supplementary Figure S5. Minimal distances between travoprost (TRV) and latanoprost (PHXA) to the K_{ATP} channel of 3 runs of each 10 µs-long coarse-grained MD simulations with the 6C3O and 6C3P structure in presence of 5 drug molecules. Each line represents the minimal distance of one drug molecule to the K_{ATP} channel.

1.2 Supplementary Tables

Supplemental Table S1. Overview of simulated systems.

	Simulation type	Structure	KATP subunits	# Drugs in simulation	Length	Replica
Betaxolol	Coarse-grained	6C3O	K_{IR}6.2 channel	10	1 µs	x10
	Atomistic	6C3O	K_{IR}6.2 channel	10	1 µs	x2
	Atomistic	6C3O	K_{IR}6.2 channel	10	350 ns	x9
Travoprost	Coarse-grained	6C3O	K_{IR}6.2 channel + 4 SUR1	5	10 µs	x3
	Coarse-grained	6C3P	K_{IR}6.2 channel + 4 SUR1	5	10 µs	x3
Latanoprost	Coarse-grained	6C3O	Kir6.2 channel + 4 SUR1	5	10 µs	x3
	Coarse-grained	6C3P	Kir6.2 channel + 4 SUR1	5	10 µs	x3

Supplemental Table S2. Parametrizations of betaxolol, travoprost and latanoprost used in the atomistic and coarse-grained MD simulations.

Topologies:

Betaxolol

(A) CG:

```
[moleculetype]

;molname   nrexcl
BXL         1

[atoms]
;id  type  resnr  residu  atom  cgnr  charge
1    SC4   1      BXL      Lin1   1     0
2    Na    1      BXL      Na1    2     0
3    SC4   1      BXL      Lin2   3     0
4    SC4   1      BXL      Ar1    4     0
5    SC4   1      BXL      Ar2    5     0
6    SC4   1      BXL      Ar3    6     0
7    Na    1      BXL      Na2    7     0
8    SC4   1      BXL      Lin3   8     0
9    SP1   1      BXL      Ohg    9     0


[ bonds ]
; Backbone bonds
    1     2      1   0.27000   20000 ;
```

```
    2     3      1   0.27000   7500 ;
    3     4      1   0.31000   20000
    4     5      1   0.42500   20000
    5     6      1   0.42500   20000
    6     7      1   0.42500   7500
    7     8      1   0.27000   20000
    8     9      1   0.31000   20000

[ constraints ]
    4     5      1   0.27000 ; PHE
    5     6      1   0.27000 ; PHE

[angles]

1   2  3  2  114.0  25.0
2   3  4  2  114.0  25.0
4   5  6  2  114.0  45.0
6   7  8  2  114.0  25.0
7   8  9  2  150.0  45.0
4   5  6  2   0     45.0
6   7  8  2   0     45.0
```

(B) atomistic:

```
; betaxolol_charged.top

;[ defaults ]
; nbfunc        comb-rule       gen-pairs       fudgeLJ fudgeQQ
;1               2               yes              0.5     0.8333

;[ atomtypes ]
;name  bond_type    mass    charge   ptype          sigma      epsilon
;ha           ha      0.0000  0.0000  A   2.59964e-01  6.27600e-02
;oh           oh      0.0000  0.0000  A   3.06647e-01  8.80314e-01
;h1           h1      0.0000  0.0000  A   2.47135e-01  6.56888e-02
;ho           ho      0.0000  0.0000  A   0.00000e+00  0.00000e+00
;hx           hx      0.0000  0.0000  A   1.95998e-01  6.56888e-02
;hc           hc      0.0000  0.0000  A   2.64953e-01  6.56888e-02
;ca           ca      0.0000  0.0000  A   3.39967e-01  3.59824e-01
;cx           cx      0.0000  0.0000  A   3.39967e-01  3.59824e-01
;os           os      0.0000  0.0000  A   3.00001e-01  7.11280e-01
;hn           hn      0.0000  0.0000  A   1.06908e-01  6.56888e-02
;c3           c3      0.0000  0.0000  A   3.39967e-01  4.57730e-01
;n4           n4      0.0000  0.0000  A   3.25000e-01  7.11280e-01

[ moleculetype ]
; Name            nrexcl
BXL             3

[ atoms ]
;   nr       type  resnr residue  atom   cgnr     charge       mass  typeB    chargeB
     1         c3      1    BXL     C1      1   -0.40613  12.000000
     2         hc      1    BXL     H1      2    0.13078   1.000000
```

```
     3         hc      1    BXL     H2      3    0.13078   1.000000
     4         hc      1    BXL     H3      4    0.13078   1.000000
     5         c3      1    BXL     C2      5    0.30738  12.000000
     6         hx      1    BXL     H4      6    0.07144   1.000000
     7         c3      1    BXL     C3      7   -0.40613  12.000000
     8         hc      1    BXL     H5      8    0.13078   1.000000
     9         hc      1    BXL     H6      9    0.13078   1.000000
    10         hc      1    BXL     H7     10    0.13078   1.000000
    11         n4      1    BXL     N1     11   -0.09657  14.000000
    12         hn      1    BXL     H8     12    0.27024   1.000000
    13         hn      1    BXL     H9     13    0.27024   1.000000
    14         c3      1    BXL     C4     14   -0.19683  12.000000
    15         hx      1    BXL    H10     15    0.13167   1.000000
    16         hx      1    BXL    H11     16    0.13167   1.000000
    17         c3      1    BXL     C5     17    0.25021  12.000000
    18         h1      1    BXL    H12     18    0.04085   1.000000
    19         oh      1    BXL     O1     19   -0.67865  16.000000
    20         ho      1    BXL    H13     20    0.46100   1.000000
    21         c3      1    BXL     C6     21    0.07907  12.000000
    22         h1      1    BXL    H14     22    0.06125   1.000000
    23         h1      1    BXL    H15     23    0.06125   1.000000
    24         os      1    BXL     O2     24   -0.36521  16.000000
    25         ca      1    BXL     C7     25    0.39688  12.000000
    26         ca      1    BXL     C8     26   -0.31428  12.000000
    27         ha      1    BXL    H16     27    0.16575   1.000000
    28         ca      1    BXL     C9     28   -0.15487  12.000000
    29         ha      1    BXL    H17     29    0.17804   1.000000
    30         ca      1    BXL    C10     30    0.02533  12.000000
    31         c3      1    BXL    C11     31   -0.14893  12.000000
    32         hc      1    BXL    H18     32    0.06540   1.000000
    33         hc      1    BXL    H19     33    0.06540   1.000000
    34         c3      1    BXL    C12     34    0.43890  12.000000
    35         h1      1    BXL    H20     35   -0.05425   1.000000
    36         h1      1    BXL    H21     36   -0.05425   1.000000
    37         os      1    BXL     O3     37   -0.44307  16.000000
    38         c3      1    BXL    C13     38    0.01917  12.000000
    39         h1      1    BXL    H22     39    0.07474   1.000000
    40         h1      1    BXL    H23     40    0.07474   1.000000
    41         cx      1    BXL    C14     41   -0.09376  12.000000
    42         hc      1    BXL    H24     42    0.14913   1.000000
    43         cx      1    BXL    C15     43   -0.33795  12.000000
    44         hc      1    BXL    H25     44    0.15994   1.000000
    45         hc      1    BXL    H26     45    0.15994   1.000000
    46         cx      1    BXL    C16     46   -0.33795  12.000000
    47         hc      1    BXL    H27     47    0.15994   1.000000
    48         hc      1    BXL    H28     48    0.15994   1.000000
    49         ca      1    BXL    C17     49   -0.15487  12.000000
    50         ha      1    BXL    H29     50    0.17804   1.000000
    51         ca      1    BXL    C18     51   -0.31428  12.000000
    52         ha      1    BXL    H30     52    0.16575   1.000000

[ bonds ]
;  ai    aj funct  r  k
    1     2     1  1.0970e-01  2.7665e+05
    1     3     1  1.0970e-01  2.7665e+05
    1     4     1  1.0970e-01  2.7665e+05
    5     6     1  1.0910e-01  2.8342e+05
    7     8     1  1.0970e-01  2.7665e+05
    7     9     1  1.0970e-01  2.7665e+05
    7    10     1  1.0970e-01  2.7665e+05
   11    12     1  1.0300e-01  3.1229e+05
   11    13     1  1.0300e-01  3.1229e+05
   14    15     1  1.0910e-01  2.8342e+05
   14    16     1  1.0910e-01  2.8342e+05
   17    18     1  1.0970e-01  2.7665e+05
   19    20     1  9.7300e-02  3.1079e+05
   21    22     1  1.0970e-01  2.7665e+05
   21    23     1  1.0970e-01  2.7665e+05
   26    27     1  1.0860e-01  2.8937e+05
   28    29     1  1.0860e-01  2.8937e+05
   31    32     1  1.0970e-01  2.7665e+05
   31    33     1  1.0970e-01  2.7665e+05
   34    35     1  1.0970e-01  2.7665e+05
```

```
   34    36     1  1.0970e-01  2.7665e+05
   38    39     1  1.0970e-01  2.7665e+05
   38    40     1  1.0970e-01  2.7665e+05
   41    42     1  1.0870e-01  2.8811e+05
   43    44     1  1.0870e-01  2.8811e+05
   43    45     1  1.0870e-01  2.8811e+05
   46    47     1  1.0870e-01  2.8811e+05
   46    48     1  1.0870e-01  2.8811e+05
   49    50     1  1.0860e-01  2.8937e+05
   51    52     1  1.0860e-01  2.8937e+05
    1     5     1  1.5380e-01  2.5179e+05
    5     7     1  1.5380e-01  2.5179e+05
    5    11     1  1.5110e-01  2.3707e+05
   11    14     1  1.5110e-01  2.3707e+05
   14    17     1  1.5380e-01  2.5179e+05
   17    19     1  1.4230e-01  2.6501e+05
   17    21     1  1.5380e-01  2.5179e+05
   21    24     1  1.4320e-01  2.5824e+05
   24    25     1  1.3700e-01  3.1514e+05
   25    26     1  1.3980e-01  3.8585e+05
   25    51     1  1.3980e-01  3.8585e+05
   26    28     1  1.3980e-01  3.8585e+05
   28    30     1  1.3980e-01  3.8585e+05
   30    31     1  1.5160e-01  2.6861e+05
   30    49     1  1.3980e-01  3.8585e+05
   31    34     1  1.5380e-01  2.5179e+05
   34    37     1  1.4320e-01  2.5824e+05
   37    38     1  1.4320e-01  2.5824e+05
   38    41     1  1.5220e-01  2.6368e+05
   41    43     1  1.5080e-01  2.7489e+05
   41    46     1  1.5080e-01  2.7489e+05
   43    46     1  1.5080e-01  2.7489e+05
   49    51     1  1.3980e-01  3.8585e+05

[ pairs ]
;  ai    aj funct
     1      8      1
     1      9      1
     1     10      1
     1     12      1
     1     13      1
     2      6      1
     2      7      1
     2     11      1
     3      6      1
     3      7      1
     3     11      1
     4      6      1
     4      7      1
     4     11      1
     5     15      1
     5     16      1
     6      8      1
     6      9      1
     6     10      1
     6     12      1
     6     13      1
     6     14      1
     7     12      1
     7     13      1
     8     11      1
     9     11      1
    10     11      1
    11     18      1
    12     15      1
    12     16      1
    12     17      1
    13     15      1
    13     16      1
    13     17      1
    14     20      1
    14     22      1
```

14	23	1
15	18	1
15	19	1
15	21	1
16	18	1
16	19	1
16	21	1
18	20	1
18	22	1
18	23	1
18	24	1
19	22	1
19	23	1
20	21	1
22	25	1
23	25	1
24	27	1
24	52	1
25	29	1
25	50	1
26	52	1
27	51	1
27	29	1
27	30	1
28	32	1
28	33	1
28	50	1
29	31	1
29	49	1
30	35	1
30	36	1
30	52	1
31	50	1
32	49	1
32	35	1
32	36	1
32	37	1
33	49	1
33	35	1
33	36	1
33	37	1
34	39	1
34	40	1
35	38	1
36	38	1
37	42	1
38	44	1
38	45	1
38	47	1
38	48	1
39	42	1
39	43	1
39	46	1
40	42	1
40	43	1
40	46	1
42	44	1
42	45	1
42	47	1
42	48	1
44	47	1
44	48	1
45	47	1
45	48	1
50	52	1
1	14	1
5	17	1
7	14	1
11	19	1
11	21	1
14	24	1
17	25	1
19	24	1

```
    21     26      1
    21     51      1
    24     28      1
    24     49      1
    25     30      1
    26     49      1
    26     31      1
    28     51      1
    28     34      1
    30     37      1
    31     51      1
    31     38      1
    34     49      1
    34     41      1
    37     43      1
    37     46      1

[ angles ]
;  ai    aj    ak funct  theta   cth
    1     5     6     1  1.1056e+02  3.8660e+02
    2     1     3     1  1.0758e+02  3.2970e+02
    2     1     4     1  1.0758e+02  3.2970e+02
    2     1     5     1  1.0980e+02  3.8744e+02
    3     1     4     1  1.0758e+02  3.2970e+02
    3     1     5     1  1.0980e+02  3.8744e+02
    4     1     5     1  1.0980e+02  3.8744e+02
    5     7     8     1  1.0980e+02  3.8744e+02
    5     7     9     1  1.0980e+02  3.8744e+02
    5     7    10     1  1.0980e+02  3.8744e+02
    5    11    12     1  1.1011e+02  3.8409e+02
    5    11    13     1  1.1011e+02  3.8409e+02
    6     5     7     1  1.1056e+02  3.8660e+02
    6     5    11     1  1.0801e+02  4.0668e+02
    8     7     9     1  1.0758e+02  3.2970e+02
    8     7    10     1  1.0758e+02  3.2970e+02
    9     7    10     1  1.0758e+02  3.2970e+02
   11    14    15     1  1.0801e+02  4.0668e+02
   11    14    16     1  1.0801e+02  4.0668e+02
   12    11    13     1  1.0830e+02  3.3974e+02
   12    11    14     1  1.1011e+02  3.8409e+02
   13    11    14     1  1.1011e+02  3.8409e+02
   14    17    18     1  1.0956e+02  3.8828e+02
   15    14    16     1  1.0975e+02  3.2803e+02
   15    14    17     1  1.1056e+02  3.8660e+02
   16    14    17     1  1.1056e+02  3.8660e+02
   17    19    20     1  1.0726e+02  3.9664e+02
   17    21    22     1  1.0956e+02  3.8828e+02
   17    21    23     1  1.0956e+02  3.8828e+02
   18    17    19     1  1.1026e+02  4.2593e+02
   18    17    21     1  1.0956e+02  3.8828e+02
   22    21    23     1  1.0846e+02  3.2803e+02
   22    21    24     1  1.0978e+02  4.2509e+02
   23    21    24     1  1.0978e+02  4.2509e+02
   25    26    27     1  1.1988e+02  4.0334e+02
   25    51    52     1  1.1988e+02  4.0334e+02
   26    28    29     1  1.1988e+02  4.0334e+02
   27    26    28     1  1.1988e+02  4.0334e+02
   29    28    30     1  1.1988e+02  4.0334e+02
   30    31    32     1  1.1047e+02  3.9162e+02
   30    31    33     1  1.1047e+02  3.9162e+02
   30    49    50     1  1.1988e+02  4.0334e+02
   31    34    35     1  1.0956e+02  3.8828e+02
   31    34    36     1  1.0956e+02  3.8828e+02
   32    31    33     1  1.0758e+02  3.2970e+02
   32    31    34     1  1.0980e+02  3.8744e+02
   33    31    34     1  1.0980e+02  3.8744e+02
   35    34    36     1  1.0846e+02  3.2803e+02
   35    34    37     1  1.0978e+02  4.2509e+02
   36    34    37     1  1.0978e+02  4.2509e+02
   37    38    39     1  1.0978e+02  4.2509e+02
   37    38    40     1  1.0978e+02  4.2509e+02
   38    41    42     1  1.1448e+02  3.8325e+02
```

```
   39    38    40     1  1.0846e+02  3.2803e+02
   39    38    41     1  1.0969e+02  3.9162e+02
   40    38    41     1  1.0969e+02  3.9162e+02
   41    43    44     1  1.1769e+02  3.8158e+02
   41    43    45     1  1.1769e+02  3.8158e+02
   41    46    47     1  1.1769e+02  3.8158e+02
   41    46    48     1  1.1769e+02  3.8158e+02
   42    41    43     1  1.1769e+02  3.8158e+02
   42    41    46     1  1.1769e+02  3.8158e+02
   43    46    47     1  1.1769e+02  3.8158e+02
   43    46    48     1  1.1769e+02  3.8158e+02
   44    43    45     1  1.1443e+02  3.2217e+02
   44    43    46     1  1.1769e+02  3.8158e+02
   45    43    46     1  1.1769e+02  3.8158e+02
   47    46    48     1  1.1443e+02  3.2217e+02
   49    51    52     1  1.1988e+02  4.0334e+02
   50    49    51     1  1.1988e+02  4.0334e+02
    1     5     7     1  1.1151e+02  5.2635e+02
    1     5    11     1  1.1421e+02  5.3723e+02
    5    11    14     1  1.0966e+02  5.2384e+02
    7     5    11     1  1.1421e+02  5.3723e+02
   11    14    17     1  1.1421e+02  5.3723e+02
   14    17    19     1  1.1019e+02  5.6484e+02
   14    17    21     1  1.1151e+02  5.2635e+02
   17    21    24     1  1.0797e+02  5.6902e+02
   19    17    21     1  1.1019e+02  5.6484e+02
   21    24    25     1  1.1796e+02  5.2300e+02
   24    25    26     1  1.1920e+02  5.8241e+02
   24    25    51     1  1.1920e+02  5.8241e+02
   25    26    28     1  1.2002e+02  5.5731e+02
   25    51    49     1  1.2002e+02  5.5731e+02
   26    25    51     1  1.2002e+02  5.5731e+02
   26    28    30     1  1.2002e+02  5.5731e+02
   28    30    31     1  1.2077e+02  5.3137e+02
   28    30    49     1  1.2002e+02  5.5731e+02
   30    31    34     1  1.1207e+02  5.2802e+02
   30    49    51     1  1.2002e+02  5.5731e+02
   31    30    49     1  1.2077e+02  5.3137e+02
   31    34    37     1  1.0797e+02  5.6902e+02
   34    37    38     1  1.1248e+02  5.2467e+02
   37    38    41     1  1.0780e+02  5.7321e+02
   38    41    43     1  1.2011e+02  5.1463e+02
   38    41    46     1  1.2011e+02  5.1463e+02
   41    43    46     1  6.0000e+01  7.3136e+02
   41    46    43     1  6.0000e+01  7.3136e+02
   43    41    46     1  6.0000e+01  7.3136e+02

[ dihedrals ]
;i  j   k  l        func     C0  ...  C5
    1    5    7    8      3     0.66944     2.00832     0.00000    -2.67776     0.00000     0.00000
         ;
    1    5    7    9      3     0.66944     2.00832     0.00000    -2.67776     0.00000     0.00000
         ;
    1    5    7    10     3     0.66944     2.00832     0.00000    -2.67776     0.00000     0.00000
         ;
    1    5    11   12     3     0.65270     1.95811     0.00000    -2.61082     0.00000     0.00000
         ;
    1    5    11   13     3     0.65270     1.95811     0.00000    -2.61082     0.00000     0.00000
         ;
    2    1    5    6      3     0.65270     1.95811     0.00000    -2.61082     0.00000     0.00000
         ;
    2    1    5    7      3     0.66944     2.00832     0.00000    -2.67776     0.00000     0.00000
         ;
    2    1    5    11     3     0.65270     1.95811     0.00000    -2.61082     0.00000     0.00000
         ;
    3    1    5    6      3     0.65270     1.95811     0.00000    -2.61082     0.00000     0.00000
         ;
    3    1    5    7      3     0.66944     2.00832     0.00000    -2.67776     0.00000     0.00000
         ;
    3    1    5    11     3     0.65270     1.95811     0.00000    -2.61082     0.00000     0.00000
         ;
    4    1    5    6      3     0.65270     1.95811     0.00000    -2.61082     0.00000     0.00000
         ;
```

4	1	5	7	3	0.66944	2.00832	0.00000	-2.67776	0.00000	0.00000	;
4	1	5	11	3	0.65270	1.95811	0.00000	-2.61082	0.00000	0.00000	;
5	11	14	15	3	0.65270	1.95811	0.00000	-2.61082	0.00000	0.00000	;
5	11	14	16	3	0.65270	1.95811	0.00000	-2.61082	0.00000	0.00000	;
6	5	7	8	3	0.65270	1.95811	0.00000	-2.61082	0.00000	0.00000	;
6	5	7	9	3	0.65270	1.95811	0.00000	-2.61082	0.00000	0.00000	;
6	5	7	10	3	0.65270	1.95811	0.00000	-2.61082	0.00000	0.00000	;
6	5	11	12	3	0.65270	1.95811	0.00000	-2.61082	0.00000	0.00000	;
6	5	11	13	3	0.65270	1.95811	0.00000	-2.61082	0.00000	0.00000	;
6	5	11	14	3	0.65270	1.95811	0.00000	-2.61082	0.00000	0.00000	;
7	5	11	12	3	0.65270	1.95811	0.00000	-2.61082	0.00000	0.00000	;
7	5	11	13	3	0.65270	1.95811	0.00000	-2.61082	0.00000	0.00000	;
8	7	5	11	3	0.65270	1.95811	0.00000	-2.61082	0.00000	0.00000	;
9	7	5	11	3	0.65270	1.95811	0.00000	-2.61082	0.00000	0.00000	;
10	7	5	11	3	0.65270	1.95811	0.00000	-2.61082	0.00000	0.00000	;
11	14	17	18	3	0.65270	1.95811	0.00000	-2.61082	0.00000	0.00000	;
12	11	14	15	3	0.65270	1.95811	0.00000	-2.61082	0.00000	0.00000	;
12	11	14	16	3	0.65270	1.95811	0.00000	-2.61082	0.00000	0.00000	;
12	11	14	17	3	0.65270	1.95811	0.00000	-2.61082	0.00000	0.00000	;
13	11	14	15	3	0.65270	1.95811	0.00000	-2.61082	0.00000	0.00000	;
13	11	14	16	3	0.65270	1.95811	0.00000	-2.61082	0.00000	0.00000	;
13	11	14	17	3	0.65270	1.95811	0.00000	-2.61082	0.00000	0.00000	;
14	17	19	20	3	1.71544	0.96232	0.00000	-2.67776	0.00000	0.00000	;
14	17	21	22	3	0.65270	1.95811	0.00000	-2.61082	0.00000	0.00000	;
14	17	21	23	3	0.65270	1.95811	0.00000	-2.61082	0.00000	0.00000	;
15	14	17	18	3	0.65270	1.95811	0.00000	-2.61082	0.00000	0.00000	;
15	14	17	19	3	0.65270	1.95811	0.00000	-2.61082	0.00000	0.00000	;
15	14	17	21	3	0.65270	1.95811	0.00000	-2.61082	0.00000	0.00000	;
16	14	17	18	3	0.65270	1.95811	0.00000	-2.61082	0.00000	0.00000	;
16	14	17	19	3	0.65270	1.95811	0.00000	-2.61082	0.00000	0.00000	;
16	14	17	21	3	0.65270	1.95811	0.00000	-2.61082	0.00000	0.00000	;
18	17	19	20	3	0.69873	2.09618	0.00000	-2.79491	0.00000	0.00000	;
18	17	21	22	3	0.65270	1.95811	0.00000	-2.61082	0.00000	0.00000	;
18	17	21	23	3	0.65270	1.95811	0.00000	-2.61082	0.00000	0.00000	;
18	17	21	24	3	1.04600	-1.04600	0.00000	0.00000	0.00000	0.00000	;
19	17	21	22	3	1.04600	-1.04600	0.00000	0.00000	0.00000	0.00000	;

19	17	21	23	3	1.04600	-1.04600	0.00000	0.00000	0.00000	0.00000	;
20	19	17	21	3	1.71544	0.96232	0.00000	-2.67776	0.00000	0.00000	;
22	21	24	25	3	1.60247	4.80742	0.00000	-6.40989	0.00000	0.00000	;
23	21	24	25	3	1.60247	4.80742	0.00000	-6.40989	0.00000	0.00000	;
24	25	26	27	3	30.33400	0.00000	-30.33400	0.00000	0.00000	0.00000	;
24	25	51	52	3	30.33400	0.00000	-30.33400	0.00000	0.00000	0.00000	;
25	26	28	29	3	30.33400	0.00000	-30.33400	0.00000	0.00000	0.00000	;
25	51	49	50	3	30.33400	0.00000	-30.33400	0.00000	0.00000	0.00000	;
26	25	51	52	3	30.33400	0.00000	-30.33400	0.00000	0.00000	0.00000	;
27	26	25	51	3	30.33400	0.00000	-30.33400	0.00000	0.00000	0.00000	;
27	26	28	29	3	30.33400	0.00000	-30.33400	0.00000	0.00000	0.00000	;
27	26	28	30	3	30.33400	0.00000	-30.33400	0.00000	0.00000	0.00000	;
28	30	31	32	3	0.00000	0.00000	0.00000	0.00000	0.00000	0.00000	;
28	30	31	33	3	0.00000	0.00000	0.00000	0.00000	0.00000	0.00000	;
28	30	49	50	3	30.33400	0.00000	-30.33400	0.00000	0.00000	0.00000	;
29	28	30	31	3	30.33400	0.00000	-30.33400	0.00000	0.00000	0.00000	;
29	28	30	49	3	30.33400	0.00000	-30.33400	0.00000	0.00000	0.00000	;
30	31	34	35	3	0.65270	1.95811	0.00000	-2.61082	0.00000	0.00000	;
30	31	34	36	3	0.65270	1.95811	0.00000	-2.61082	0.00000	0.00000	;
30	49	51	52	3	30.33400	0.00000	-30.33400	0.00000	0.00000	0.00000	;
31	30	49	50	3	30.33400	0.00000	-30.33400	0.00000	0.00000	0.00000	;
32	31	30	49	3	0.00000	0.00000	0.00000	0.00000	0.00000	0.00000	;
32	31	34	35	3	0.65270	1.95811	0.00000	-2.61082	0.00000	0.00000	;
32	31	34	36	3	0.65270	1.95811	0.00000	-2.61082	0.00000	0.00000	;
32	31	34	37	3	1.04600	-1.04600	0.00000	0.00000	0.00000	0.00000	;
33	31	30	49	3	0.00000	0.00000	0.00000	0.00000	0.00000	0.00000	;
33	31	34	35	3	0.65270	1.95811	0.00000	-2.61082	0.00000	0.00000	;
33	31	34	36	3	0.65270	1.95811	0.00000	-2.61082	0.00000	0.00000	;
33	31	34	37	3	1.04600	-1.04600	0.00000	0.00000	0.00000	0.00000	;
34	37	38	39	3	1.60247	4.80742	0.00000	-6.40989	0.00000	0.00000	;
34	37	38	40	3	1.60247	4.80742	0.00000	-6.40989	0.00000	0.00000	;
35	34	37	38	3	1.60247	4.80742	0.00000	-6.40989	0.00000	0.00000	;
36	34	37	38	3	1.60247	4.80742	0.00000	-6.40989	0.00000	0.00000	;
37	38	41	42	3	1.04600	-1.04600	0.00000	0.00000	0.00000	0.00000	;
38	41	43	44	3	0.65270	1.95811	0.00000	-2.61082	0.00000	0.00000	;
38	41	43	45	3	0.65270	1.95811	0.00000	-2.61082	0.00000	0.00000	;

38	41	46	47	3	0.65270	1.95811	0.00000	-2.61082	0.00000	0.00000	;
38	41	46	48	3	0.65270	1.95811	0.00000	-2.61082	0.00000	0.00000	;
39	38	41	42	3	0.62760	1.88280	0.00000	-2.51040	0.00000	0.00000	;
39	38	41	43	3	0.66944	2.00832	0.00000	-2.67776	0.00000	0.00000	;
39	38	41	46	3	0.66944	2.00832	0.00000	-2.67776	0.00000	0.00000	;
40	38	41	42	3	0.62760	1.88280	0.00000	-2.51040	0.00000	0.00000	;
40	38	41	43	3	0.66944	2.00832	0.00000	-2.67776	0.00000	0.00000	;
40	38	41	46	3	0.66944	2.00832	0.00000	-2.67776	0.00000	0.00000	;
41	43	46	47	3	0.65270	1.95811	0.00000	-2.61082	0.00000	0.00000	;
41	43	46	48	3	0.65270	1.95811	0.00000	-2.61082	0.00000	0.00000	;
41	46	43	44	3	0.65270	1.95811	0.00000	-2.61082	0.00000	0.00000	;
41	46	43	45	3	0.65270	1.95811	0.00000	-2.61082	0.00000	0.00000	;
42	41	43	44	3	0.65270	1.95811	0.00000	-2.61082	0.00000	0.00000	;
42	41	43	45	3	0.65270	1.95811	0.00000	-2.61082	0.00000	0.00000	;
42	41	43	46	3	0.65270	1.95811	0.00000	-2.61082	0.00000	0.00000	;
42	41	46	43	3	0.65270	1.95811	0.00000	-2.61082	0.00000	0.00000	;
42	41	46	47	3	0.65270	1.95811	0.00000	-2.61082	0.00000	0.00000	;
42	41	46	48	3	0.65270	1.95811	0.00000	-2.61082	0.00000	0.00000	;
43	41	46	47	3	0.65270	1.95811	0.00000	-2.61082	0.00000	0.00000	;
43	41	46	48	3	0.65270	1.95811	0.00000	-2.61082	0.00000	0.00000	;
44	43	41	46	3	0.65270	1.95811	0.00000	-2.61082	0.00000	0.00000	;
44	43	46	47	3	0.65270	1.95811	0.00000	-2.61082	0.00000	0.00000	;
44	43	46	48	3	0.65270	1.95811	0.00000	-2.61082	0.00000	0.00000	;
45	43	41	46	3	0.65270	1.95811	0.00000	-2.61082	0.00000	0.00000	;
45	43	46	47	3	0.65270	1.95811	0.00000	-2.61082	0.00000	0.00000	;
45	43	46	48	3	0.65270	1.95811	0.00000	-2.61082	0.00000	0.00000	;
50	49	51	52	3	30.33400	0.00000	-30.33400	0.00000	0.00000	0.00000	;
25	28	26	27	3	9.20480	0.00000	-9.20480	0.00000	0.00000	0.00000	;
26	30	28	29	3	9.20480	0.00000	-9.20480	0.00000	0.00000	0.00000	;
30	51	49	50	3	9.20480	0.00000	-9.20480	0.00000	0.00000	0.00000	;
25	49	51	52	3	9.20480	0.00000	-9.20480	0.00000	0.00000	0.00000	;
1	5	11	14	3	0.65270	1.95811	0.00000	-2.61082	0.00000	0.00000	;
5	11	14	17	3	0.65270	1.95811	0.00000	-2.61082	0.00000	0.00000	;
7	5	11	14	3	0.65270	1.95811	0.00000	-2.61082	0.00000	0.00000	;
11	14	17	19	3	0.60250	1.80749	10.87840	-2.40998	0.00000	0.00000	;
11	14	17	21	3	0.65270	1.95811	0.00000	-2.61082	0.00000	0.00000	;

```
    14   17   21   24     3     0.65270     1.95811     0.00000    -2.61082     0.00000     0.00000
         ;
    17   21   24   25     3     1.60247     4.80742     0.00000    -6.40989     0.00000     0.00000
         ;
    19   17   21   24     3     0.60250     1.80749     9.83240    -2.40998     0.00000     0.00000
         ;
    21   24   25   26     3     7.53120     0.00000    -7.53120     0.00000     0.00000     0.00000
         ;
    21   24   25   51     3     7.53120     0.00000    -7.53120     0.00000     0.00000     0.00000
         ;
    24   25   26   28     3    30.33400     0.00000   -30.33400     0.00000     0.00000     0.00000
         ;
    24   25   51   49     3    30.33400     0.00000   -30.33400     0.00000     0.00000     0.00000
         ;
    25   26   28   30     3    30.33400     0.00000   -30.33400     0.00000     0.00000     0.00000
         ;
    25   51   49   30     3    30.33400     0.00000   -30.33400     0.00000     0.00000     0.00000
         ;
    26   25   51   49     3    30.33400     0.00000   -30.33400     0.00000     0.00000     0.00000
         ;
    26   28   30   31     3    30.33400     0.00000   -30.33400     0.00000     0.00000     0.00000
         ;
    26   28   30   49     3    30.33400     0.00000   -30.33400     0.00000     0.00000     0.00000
         ;
    28   26   25   51     3    30.33400     0.00000   -30.33400     0.00000     0.00000     0.00000
         ;
    28   30   31   34     3     0.00000     0.00000     0.00000     0.00000     0.00000     0.00000
         ;
    28   30   49   51     3    30.33400     0.00000   -30.33400     0.00000     0.00000     0.00000
         ;
    30   31   34   37     3     0.65270     1.95811     0.00000    -2.61082     0.00000     0.00000
         ;
    31   30   49   51     3    30.33400     0.00000   -30.33400     0.00000     0.00000     0.00000
         ;
    31   34   37   38     3     2.43927     4.80742    -0.83680    -6.40989     0.00000     0.00000
         ;
    34   31   30   49     3     0.00000     0.00000     0.00000     0.00000     0.00000     0.00000
         ;
    34   37   38   41     3     1.60247     4.80742     0.00000    -6.40989     0.00000     0.00000
         ;
    37   38   41   43     3     0.65270     1.95811     0.00000    -2.61082     0.00000     0.00000
         ;
    37   38   41   46     3     0.65270     1.95811     0.00000    -2.61082     0.00000     0.00000
         ;
    38   41   43   46     3     0.65270     1.95811     0.00000    -2.61082     0.00000     0.00000
         ;
    38   41   46   43     3     0.65270     1.95811     0.00000    -2.61082     0.00000     0.00000
         ;
    26   51   25   24     3     9.20480     0.00000    -9.20480     0.00000     0.00000     0.00000
         ;
    28   49   30   31     3     9.20480     0.00000    -9.20480     0.00000     0.00000     0.00000
         ;

;[ system ]
;52 system

;[ molecules ]
; Compound        nmols
;solute            1
```

Coarse-grained topology of travoprost:

```
[moleculetype]

;molname   nrexcl

TRV         1

[atoms]
```

```
;id  type  resnr  residu  atom  cgnr  charge
1    SC4   1      TRV      CF3    1     0
2    SC4   1      TRV      AR1    2     0
3    SC4   1      TRV      AR2    3     0
4    SC4   1      TRV      AR3    4     0
5    P1    1      TRV      OXOH   5     0
6    SC3   1      TRV      C=C1   6     0
7    SP1   1      TRV      R1     7     0
8    SP1   1      TRV      R2     8     0
9    C3    1      TRV      C=C2   9     0
10   SC1   1      TRV      C-C1   10    0
11   SNa    1      TRV      OXO    11    0
12   SC1   1      TRV      C-C2   12    0

[ bonds ]
    4     5      1   0.3200  100000
    5     6      1   0.3100  80000
    6     7      1   0.2950  50000
    6     8      1   0.3400  50000
    7     8      1   0.2500  10000
    8     9      1   0.3400  5000
    9    10      1   0.3200  5000
    ;10   11      1   0.2350  100000
    ;11   12      1   0.2500  100000


[ constraints ]
    1     2      1   0.24
    2     3      1   0.27
    3     4      1   0.27
    2     4      1   0.27
    10   11      1   0.235
    11   12      1   0.25


[angles]
    1   2   3    1    100.0   1000.0 ;
    3   4   5    1    160.0   60.0
    4   5   6    1    120.0   30.0
    5   6   7    1    160.0   50.0
    6   8   9    1    75.0    800.0 ;
    7   8   9    1    130.0   80.0
    8   9   10   1    140.0   20.0
```

```
    9   10  11   1    120.0   10.0 ;
    10  11  12   1    155.0   300.0;


[ dihedrals ]
   ;ai   aj   ak   al   type  phi  kfac
    1    2    3    4    2      180   500.0
    ;2    3    4    5    1      0     10.0  1
    ;4    5    6    7    1      180   10.0  1
    5    6    7    8    1      0   2.0   1
    8    9    10   11   1      0   2.0   1
```

Coarse-grained topology of latanoprost:

```
[moleculetype]

;molname   nrexcl
PHX         1

[atoms]
;id  type  resnr  residu  atom  cgnr  charge
1    SC1   1      PHX      AC1    1     0
2    SNa   1      PHX      COO    2     0
3    SC1   1      PHX      AC2    3     0
4    SC3   1      PHX      DB     4     0
5    P1    1      PHX      R1     5     0
6    SP1   1      PHX      R2     6     0
7    SP1   1      PHX      R3     7     0
8    P1    1      PHX      OH     8     0
9    SC1   1      PHX      AC3    9     0
10   SC4   1      PHX      R4     10    0
11   SC4   1      PHX      R5     11    0
12   SC4   1      PHX      R6     12    0

[ bonds ]
    ;1     2      1   0.250  30000
    2     3      1   0.280  10000
    3     4      1   0.280  4000
    ;4     5      1   0.2450 20000
    5     6      1   0.2400 50000
    6     7      1   0.2400 5000
    5     7      1   0.3000 15000
    7     8      1   0.3650 10000
    8     9      1   0.2850 10000
    9     10     1   0.22   10000



[ constraints ]
    1     2      1   0.25
    4     5      1   0.2450
    10    11      1   0.27
    11    12      1   0.27
    10    12      1   0.27
```

```
[angles]
    1   2   3    1    155.0   100.0 ;
    2   3   4    1    140.0   10.0
    3   4   5    1    100.0   30.0
    ;4   5   6    1    120.0   50.0 ;
    5   6   7    1    80.0    600.0
    6   7   8    1    130.0   300.0 ;
    7   8   9    1    140.0   40.0
    ;8   9   10   1    160.0   20.0
    9   10   11   1    155.0  400.0
    ;10  11  12   1    150.0   80.0


[ dihedrals ]
   ;ai   aj   ak   al   type  phi    kfac   multipl
     ;8    9   10   11    1     0     10.0   1
     5    6    7    8    1     25    10.0   1
```

Supplemental Table S3. Details of travoprost (TRV) and latanoprost (PHXA) interaction sites found in the coarse-grained MD simulations.

6C3O – Details of binding sites found in cg MD simulations

Cluster	# ligands in cluster TRV	# ligands in cluster PHXA	Interacting residues over last 100 ns
1	2	3	D159, Y453, S459, I462, F545, I557, F565, M1065, V1066, T1068, S1072
2		2	A315, G316, L318, F321, V357, L358, L361, L362, L364, A365 , Y454, I455, L582
3	2	3	W288,L438,W439 ,Q474,Y475,T479,N547,L586,L590,T600, N1480,F1481,S1482
4	1		F79, F83, L174, Y356, F363, Y1253
5	1	2	W51, Q219, F221, N223, W997, Y1003, T1088, S1143, T1146, Y1175
6		1	F217, Y378, F1239, W1246
7	3		Q273, T281, N397, H401, S611, S615
8	2	2	Q664, S665, C677, Q680, Q714, F916, Q922, Q1458, N1480, Q1485, T1515
9	1	2	T695, S697, H895, A901 , F916, Q922, W92, Q934, T1495, F1527, T1534, T1541
10	1		P1331, W1338, D1408, L1411, Y1416
11	2		V667, M682, F738, P809, S956, R957, S960, S961, T1380, V1550, F1576

6C3P – Details of binding sites found in cg MD simulations

Cluster	# ligands in cluster TRV	# ligands in cluster PHXA	Interacting residues over last 100 ns
1	1	4	V560, F565, F566, Q568, T1068, S1072, L1079
2	1		F27, V34, F35, F38
3	1	2	F114, Y356, F1163, V1165, A1166, T1265, S1268
4	4		F83, S95, S165, L171, L312, F321, H326, Y344, S347, N354, L361, Q369, F321
5		1	W997, Q1019, S1143
6		2	W232, F236, G251, N262, L266, A269, F270, Q273, L291, F295, I391, K394, S611, L614, S615, Y1229, S1232
7	3		N262, L266, A269 ,F270, Q273, L291, F295, K394, S611, S615
8	1	1	F68, F221, T261 ,F295, T304, T383, N386
9		1	R486, N493, V605, Q616
10		4	S1324, I1334, P1335, W1338, Q1341, G1342, F1391, I1407, Y1416, L1417, T1531
11	3		T687, W688, T695, Q714, T906, N1480, S1482, Q1485, T1515
12	1		F518, T524, G1433, F1437
